# MiR-585-5p impedes gastric cancer proliferation and metastasis by orchestrating the interactions among CREB1, MAPK1 and MITF

**DOI:** 10.3389/fimmu.2022.1008195

**Published:** 2022-10-04

**Authors:** Yunwei Wang, Ming Li, Jiaoxia Zeng, Yunshu Yang, Zengshan Li, Sijun Hu, Fangfang Yang, Na Wang, Wenlan Wang, Jun Tie

**Affiliations:** ^1^ State key Laboratory of Cancer Biology and Xijing Hospital of Digestive Diseases, Xijing Hospital, Air Force Medical University, Xi’an, China; ^2^ Department of Burns and Cutaneous Surgery, Xijing Hospital, Air Force Medical University, Xi’an, China; ^3^ Department of Gastroenterology, Xi’an People’s Hospital (Xi’an Fourth Hospital), Xi’an, China; ^4^ Department of Pathology, Xijing Hospital, Air Force Medical University, Xi’an, China; ^5^ Department of Aerospace Hygiene, School of Aerospace Medicine, Air Force Medical University, Xi’an, China

**Keywords:** MITF, CREB1, MAPK1, MiR-585-5p, Gastric Cancer

## Abstract

**Background:**

Gastric cancer (GC) is one of the most malignant and lethal cancers worldwide. Multiple microRNAs (miRNAs) have been identified as key regulators in the progression of GC. However, the underlying pathogenesis that miRNAs govern GC malignancy remains uncertain. Here, we identified a novel miR-585-5p as a key regulator in GC development.

**Methods:**

The expression of miR-585-5p in the context of GC tissue was detected by *in situ* hybridization for GC tissue microarray and assessed by H-scoring. The gain- and loss-of-function analyses comprised of Cell Counting Kit-8 assay and Transwell invasion and migration assay. The expression of downstream microphthalmia-associated transcription factor (MITF), cyclic AMP-responsive element-binding protein 1 (CREB1) and mitogen-activated protein kinase 1 (MAPK1) were examined by Immunohistochemistry, quantitative real-time PCR and western blot. The direct regulation between miR-585-5p and MITF/CREB1/MAPK1 were predicted by bioinformatic analysis and screened by luciferase reporter assay. The direct transcriptional activation of CREB1 on MITF was verified by luciferase reporter assay, chromatin immunoprecipitation (ChIP) and electrophoretic mobility shift assays (EMSAs). The interaction between MAPK1 and MITF was confirmed by co-immunoprecipitation (Co-IP) and immunofluorescent double-labelled staining.

**Results:**

MiR-585-5p is progressively downregulated in GC tissues and low miR-585-5p levels were strongly associated with poor clinical outcomes. Further gain- and loss-of-function analyses showed that miR-585-5p possesses strong anti-proliferative and anti-metastatic capacities in GC. Follow-up studies indicated that miR-585-5p targets the downstream molecules CREB1 and MAPK1 to regulate the transcriptional and post-translational regulation of MITF, respectively, thus controlling its expression and cancer-promoting activity. MiR-585-5p directly and negatively regulates MITF together with CREB1 and MAPK1. According to bioinformatic analysis, promotor reporter gene assays, ChIP and EMSAs, CREB1 binds to the promotor region to enhance transcriptional expression of MITF. Co-IP and immunofluorescent double-labelled staining confirmed interaction between MAPK1 and MITF. Protein immunoprecipitation revealed that MAPK1 enhances MITF activity *via* phosphorylation (Ser73). MiR-585-5p can not only inhibit MITF expression directly, but also hinder MITF expression and pro-cancerous activity in a CREB1-/MAPK1-dependent manner indirectly.

**Conclusions:**

In conclusion, this study uncovered miR-585-5p impedes gastric cancer proliferation and metastasis by orchestrating the interactions among CREB1, MAPK1 and MITF.

## Introduction

GC (GC) is a highly malignant and lethal malignancy worldwide, having over 1 million estimated novel cases annually (1). Although advances were achieved for early diagnosis and therapy in GC, cases of unresectable GC are limited to life-prolonging palliative care options (2). Moreover, the underlying mechanism that governs GC malignancy remains uncertain. Hence, it’s urgent to explore the intrinsic molecular mechanism to find new effective therapeutic targets.

MicroRNAs (miRNAs), a family of critical small non-coding RNAs, directly bind onto 3’-untranslated regions (3’UTRs) of designated transcripts, leading to translational inhibition or obliteration, thus endowing miRNAs with an crucial role in regulating multiple biological processes (3). Multiple miRNAs were found to be essential regulators within GC progression. In particular, we previously discovered that miR-218-5p is highly downregulated within GC tissue-types and displays pivotal inhibition of oncogenesis and the development of GC (4, 5). The gene encoding miR-218-5p is situated within intron of SLIT2 and SLIT3 (6), and the miR-585 gene was delineated within same region based on gene cluster analysis. As different miRNAs affiliated with the same gene cluster mostly display tightly synchronized expression and similar functions (7), we speculate that miR-585-5p might be important for regulating malignancy of GC. Nonetheless, the role of miR-585-5p in tumour pathogenesis is rarely defined. Recently, reports revealed that miR-585 is downregulated and acts as a tumour-suppressive miRNA within colon cancer, non-small-cell lung cancer, glioma and GC (8–11). Hu et al. (11) confirmed that miR-585 downregulation is linked with invasion/TNM stage/lymph node invasion levels and poor prognosis levels. However, the potential role and intrinsic mechanisms governing miR-585-5p function are poorly understood. Microphthalmia-associated transcription factor (MITF) was parsed out as a core-target based on target prediction of miR-585-5p. MITF, a melanocytic lineage-specific transcription factor, has been certified as a master regulator of melanocyte homeostasis and melanoma progression (12). However, the biology of MITF in GC requires further research.

This investigation highlighted that miR-585-5p is downregulated within GC and is associated with poor prognosis, revealing miR-585-5p influence upon suppressing GC proliferative/metastatic properties. Furthermore, this investigation demonstrated that MITF had a straight positive regulation of GC proliferative/metastatic properties. CREB1 activates MITF transcription, and MAPK1 enhances the activity of MITF via phosphorylation at serine 73, boosting the cancer-promoting effect of MITF in GC. MiR-585-5p directly simultaneously inhibits expression of MITF, CREB1 and MAPK1 in a post-transcriptional manner. Consequently, miR-585-5p also indirectly restrains MITF transcription and activity through directly inhibiting the expression of CREB1 and MAPK1. Overall, we report for the first time that miR-585-5p suppresses GC proliferative/metastatic properties by orchestrating the interactions among CREB1, MAPK1 and MITF.

## Materials and methods

### Clinical samples

The GC tissue microarray for in situ hybridization (ISH) of miR-585-5p was purchased from Shanghai Outdo Biotech: HStmA180Su15 contains 16 cases of unpaired GC tissue-types and 82 cases of paired gastric adenocarcinoma and paraneoplastic tissue-types with one point for each tissue, all with long-term clinical follow-up records. The GC tissue microarray for immunohistochemistry of MITF was purchased from Avilabio: DC-Sto11020 contains 10 unpaired normal tissue-types and 45 paired GC and paraneoplastic tissue-types.

### Immunohistochemistry (IHC) and ISH

IHC and ISH staining were performed to quantify expression of the MITF protein and miR-585-5p, respectively. GC tissue microarrays were immuno-stained for MITF (Abcam, # ab270262). The digoxin-labelled nucleic acid probe for miR-585-5p was developed through GenePharma using the reverse complement of the following sequence: 25-CUAGCACACAGAUACGCCCAGA-46. The microarrays were observed/imaged through Pannoramic 250FLASH Scanner (3DHISTECH). IHC and ISH staining were concomitantly assessed through two blinded observers for individual clinical case clinico-pathological profiles. H-scoring was adopted based upon intensity and extent of staining by an experienced pathologist, and graded as: 0, negative staining; 1+, weak staining; 2+, moderate staining; and 3+, strong staining. The H-score was computed by multiplying the different intensities in 4 gradations with each percentage of positive tumour cells: H-score = 1× (% cells 1+) + 2× (% cells 2+) + 3× (% cells 3+). Finally, a score from 0 to 300 points was obtained (13). Median H-score within cohort was applied as a cut-off for distinguishing high- and low-expression.

### Transient transfection and lentivirus infection

Bioengineered novel recombinant miR-585-5p mimics, inhibitors and complementing negative controls were procured through Rongqingchang Biotech (China). Cultures including AGS, BGC823 and HGC27 lines at a confluence of 50% were exposed to transfection with miR-585-5p mimics or inhibitors employing Lipofectamine 3000^®^ reagent (Invitrogen™, # L3000015) in line with kit protocols, with mimics-NC or inhibitors-NC, respectively, as controls. Ectopic expression efficiency tests and follow-up experiments were conducted at 48 h following transfection.

Lentivirus expression plasmids for MITF-overexpression, MITF-mutant (S73A), shMITF (Target sequence of shMITF-3: GGTGAATCGGATCATCAAG), CREB1-overexpression, shCREB1 (Target sequence of shCREB1-3: acATTAGCCCAGGTATCTATG), MAPK1-overexpression and shMAPK1 (Target sequence: caAAGTTCGAGTAGCTATCAA) were constructed by GeneChem™ (Shanghai, China). Target cultures were exposed to 1×10^7^ lentivirus transducing units within presence of HitransG P reagents (GeneChem, #REVG005, 1:25). Homologous empty lentiviral vectors acted as negative controls. The cultures were employed following infection and antibiotic selection for 4 weeks.

### *In vivo* tumorigenicity

All animals in this investigation were purchased from the Experimental Animal Center of Fourth Military Medical University. All procedures were conducted in line with ARRIVE guidelines and accepted by the Ethics Committee of Fourth Military Medical University. Parental BGC823 cultures (5×10^5^ cultures in 200 μl of PBS) were subcutaneously inoculated within ventral flank of 6-week-old male Balb/c nude murines (ten murines/cohort). Tumour diameter was quantified every two days, and following successful establishment of orthotopic xenograft tumorigenicity on the 13^th^ day (the tumour diameters reached approximately 3-5 mm), 10 μg of miR-585-5p mimics or normal saline or the negative control was introduced within tumours every 48h for 2 weeks. Ten micrograms of mimics and 1.2 μl of *in vivo* jetPEI reagents (Polyplus Transfection, #PT-201-50G) were dissolved in 12.5 μl of 10% glucose, and sterile water was added to 25 μl. Ultimately, the two were incubated to form the internal delivery system. Twenty-nine days following subcutaneous tumour injection, all murines were sacrificed, and all tumours were removed, weighed, harvested and paraffin-embedded. Tumour volume (mm^3^) was determined depending upon longest/shortest diameters as: 


$$\text{volume}={\left(\text{shortest diameter}\right)}^{2}\times \left(\text{longest diameter}\right)\times 0.5.$$

### Chromatin immunoprecipitation

A SimpleChIP^®^ Enzymatic Chromatin IP Kit^®^ (Cell Signaling Technology™, #9003) was employed for conducting ChIP assay. About 1×10^7^ HGC27 cultures were cross-linked within 1% formaldehyde (Fuyu Fine, Tianjin), at room temperature for 15 minutes. The nuclear protein was extracted in 1× ChIP buffer within 1 M DDT (dithiothreitol) in line with kit instructions. Micrococcal Nuclease (Cell Signaling Technology, #10011) was used for digesting DNA into fragments of 150-900 bp. Following ultrasonication, the supernatants containing cross-linked chromatin were collected by centrifugation at 9,400×g and consequently placed into incubation with anti-CREB1 antibodies at 4°C with gentle rotation overnight. Equal amounts of Normal Rabbit IgG (Cell Signaling Technology™, #2729) and Histone H3 (D2B12) XP^®^ Rabbit mAb (Cell Signaling Technology™, #4620) acted as the negative control and positive control, accordingly. ChIP-Grade Protein G Magnetic Beads^®^ (Cell Signaling Technology™, #9006) were added the next day for 2-hour incubation with gentle rotation. Following the bead-antibody complexes were washed employing 1× ChIP buffer for 3 times on the DynaMag™-2 Magnetic Separation Rack (Invitrogen™, #12321D), the complexes were eluted and de-crosslinked with 5 mg/mL Proteinase K and 5 M NaCl and at 65°C for 2 h. The DNA was subsequently purified and subjected to PCR with the MITF promoter primers MITF ChIP Forward, AGAACTCCAGCCCTAACATC, and Reverse, TCTCATTTTGGTGTTTGGCC.

### Electrophoretic mobility shift assay

EMSA was employed for determining direct binding for CREB1 protein to the MITF DNA promotor *in vitro* employing LightShift™ Chemiluminescent EMSA Kit (Thermo Fisher, #20148). A prokaryotic vector for CREB1 expression was transformed into BL21 *Escherichia coli* and driven through isopropyl-beta;-d-thiogalactoside (IPTG). Bacterial pellets were lysed, and the recombinant CREB1 protein was purified through Ni-chelating affinity chromatography. Double-stranded DNA probes were synthesized employing the following sequences: MITF-wt-FF (5`6-FAM(FITC)-fluorescently labelled primers forward), TGGATGTCTTTTCTGATGTGAAATTAAA; MITF-wt-R (unlabelled primers reverse), TTTAATTTCACATCAGAAAAGACATCCA; MITF-mut-FF, TGGATGTCTTTCTCAGCATGAAATTAAA; MITF-mut-R, TTTAATTTCATGCTGAGAAAGACATCCA. The binding reactions, including 1 μl of ddH_2_O, 2 μl of binding buffer (5×), 6 μl of recombinant CREB1 protein and 1 μl of labelled-MITF-wt probes or labelled-MITF-mut probes, proceeded at 25°C for 20 min. The reaction products were added to 1 μl of EMSA/gel-shift loading buffer and segregated through SDS-PAGE. The gel was exposed and photographed employing an FLA-9000 apparatus (FujiFilm, Japan).

### Immunoprecipitation

HGC27 cell pellets were lysed within IP lysis buffer (Beyotime, #P0013) harboring protease inhibitors (Boster, #AR1182-1) for 1 h with gentle rotation at 4°C. Supernatant lysates were collected by centrifuging at 12,000 rpm/min. Normal IgG antibody and protein A/G magnetic beads (Thermo Scientific™, #88802) acted for preclearing to lessen nonspecific binding, and then the lysates were incubated with the indicated antibodies. Equivalent protein lysates were premixed with an anti-flag antibody, an anti-HA antibody, or a normal rabbit IgG antibody as the negative control with gentle rotation at 4°C for 120 minutes and then placed into incubation with protein A/G magnetic beads (Thermo Scientific™, #88802) overnight. Resultant complexes were twice-washed with IP lysis buffer and three times with PBS, and the beads were resuspended in an equal volume of 2 × loading buffer and denatured at 100°C for 10 minutes on heat blocks, subsequently subjected to western blot analysis.

### Statistical analysis

GraphPad Prism 8.0^®^ software was employed in this case. All quantitative data-points reflected mean ± SEM. Statistical significance among multiple cohort analyses were identified through Analysis of variance (ANOVA) with Tukey’s *post hoc* test. Student’s t-test was employed for comparing mean variables of two cohorts. Kaplan-Meier method and log-rank t-test for significance were employed for survival analysis.

Detailed information is listed within Appendix S1.

## Results

### MiR-585-5p is markedly downregulated in human GC tissue-types, predicting poor prognoses

**Figure 1:**
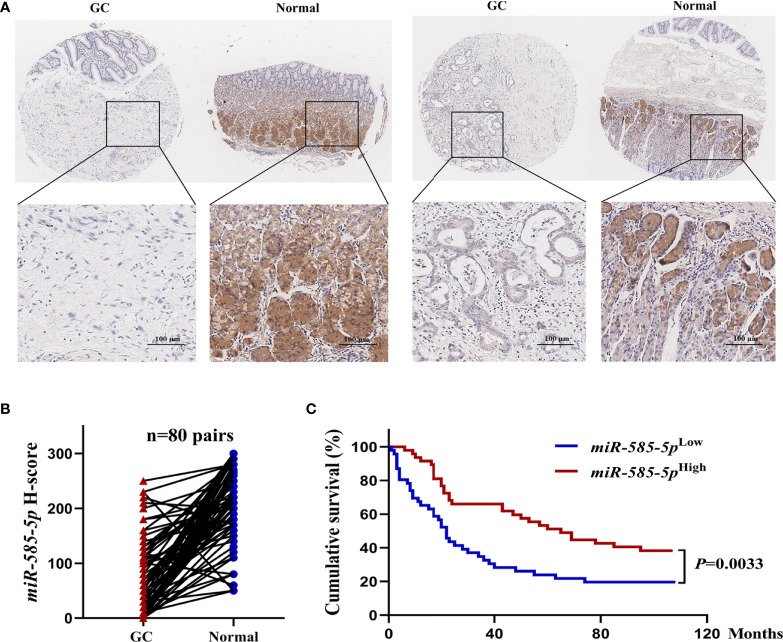
MiR-585-5p is markedly down-regulated within human GC tissue-types, predicting poor prognoses. **(A)** *In situ* hybridization of miR-585-5p within GC tissue/paired paraneoplastic tissue-types on a set of tissue microarray samples. Representative images of miR-585-5p within GC/paired paraneoplastic tissue-types. **(B)** Quantitative analyses for miR-585-5p expression within 80 paired GC and adjacent non-tumour tissue-types. MiR-585-5p expression levels were identified through multiplying staining intensity score value with score value of positive region. **(C)** Kaplan-Meier curves highlighting overall survival for GC cases (n = 93) based on different levels of miR-585-5p expression. Scale bar = 100 μm.

In order to quantify miR-585-5p expression-profile within GC, it was examined in a set of tissue microarray samples employing *in situ* hybridization. Compared with para-cancerous tissue-types, miR-585-5p expression in primary GC tissue-types was severely downregulated, and staining showed that miR-585-5p was mostly located within cytoplasm of adenocytes in gastric glands (**Figure 1A**). ISH quantitative analysis of 80 pairs of GC tissue-types and neighboring healthy tissue-types revealed a clear reduction in miR-585-5p levels in GC (**Figure 1B**). Based on quantitative analysis of miR-585-5p levels by H-scoring, 93 cases of GC (HStmA180Su15) were segregated within high miR-585-5p expression (n=47) and low miR-585-5p expression (n=46) cohorts, and correlation across miR-585-5p level and overall survival was analysed. Kaplan-Meier analyses showed that cases of positive miR-585-5p expression had prolonged overall survival (**Figure 1C**). Such dataset outcomes suggest that miR-585-5p might participate within GC carcinogenesis/development.

### MiR-585-5p suppresses the proliferative/metastatic properties of GC at multiple levels

**Figure 2:**
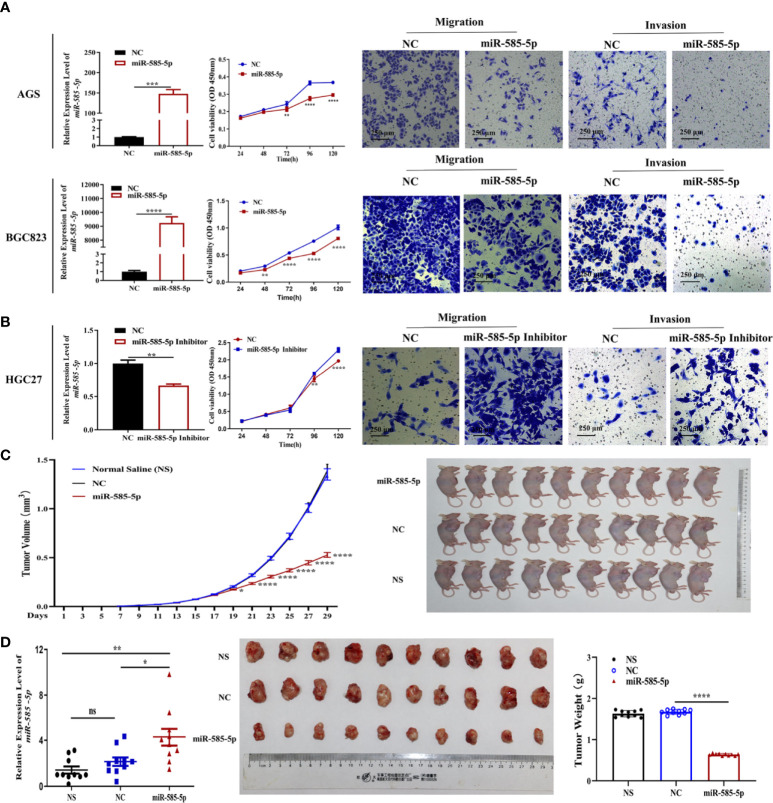
MiR-585-5p suppresses the proliferative/metastatic properties of GC *at multiple levels*. AGS and BGC823 cultures were exposed to transfection with 100 nM miR-585-5p mimics or negative control mimics, and HGC27 cultures were exposed to transfection with 100 nM miR-585-5p inhibitor or negative control. **(A, B)** MiR-585-5p levels were analysed by RT-qPCR following transfection of miR-585-5p in AGS and BGC823 cultures as well as inhibitor in HGC27 cultures. CCK-8 assays were performed to examine proliferation post transfection. Metastasis ability was evaluated by migration and invasion assays. CCK-8 and Transwell assays indicated that miR-585-5p highly suppresses the proliferative/metastatic properties of GC cultures. **(C)** BGC823 cultures were inoculated into nude murines subcutaneously. Following successful establishment of xenograft tumorigenicity on the 13^th^ day, miR-585-5p mimics, normal saline or the negative control were introduced within tumours twice a day for two weeks (n=10). MiR-585-5p remarkably hindered stomach tumour growth *in vivo*. **(D)** Both the volume and weight of the orthotopic xenograft tumours were highly smaller within miR-585-5p cohort than within control cohort. Data are presented as the mean ± SEM. *P < 0.05, **P < 0.01 , ***P < 0.001 and ****P < 0.0001 in comparison with the NC cohort. Scale bar = 250 μm. ns indicates no significance.

We assessed miR-585-5p expression-profile within multiple GC cell lines, with dataset outcomes demonstrating comparatively low levels of miR-585-5p in AGS and BGC823 cultures but obviously high expression in HGC27 cultures (**Supplementary Figure 1**). Consequently, loss-of-function experiments to validate miR-585-5p were carried out employing HGC27 cultures and gain-of-function experiments in AGS and BGC823 cultures. MiR-585-5p mimics and inhibitors were utilized to overexpress and knockdown miR-585-5p in GC cultures. Following transient transfection for 48 h, the level of miR-585-5p was increased in AGS and BGC823 cultures and downregulated in HGC27 cultures (**Figures 2A, B**). Along with the enhancement of miR-585-5p, cell viability declined highly. Transwell assays showed that miR-585-5p upregulation remarkably decreased the migration and invasion of AGS and BGC823 cultures *in vitro* (**Figure 2A**). Conversely, inhibition of miR-585-5p dramatically increased cell proliferative, migrative and invasive effects in HGC27 cultures compared with negative controls (**Figure 2B**). Furthermore, in nude murines with subcutaneous inoculation of BGC823 cultures, tumour growth as well as both the volume and weight of orthotopic xenograft tumours were highly reduced with miR-585-5p intra-tumour therapy, in contrast to normal saline or negative controls (**Figures 2C, D**). In summary, miR-585-5p plays a pivotal part in regulating the proliferative/metastatic properties of GC at multiple levels.

### MiR-585-5p directly inhibits MITF expression

The intrinsic mechanism by which miR-585-5p acts in GC development remains to be elucidated. One of the most important modes of miRNA functioning is miRNA-mediated post-transcriptional mRNA transcript repression *via* binding to 3’UTRs (14). To uncover the exact antitumour mechanism of miR-585-5p in GC, miRWalk 3.0 (http://mirwalk.umm.uni-heidelberg.de/) was employed for predicting target-genes. MITF-3’UTR was found to contain miR-585-5p-binding sites, indicating MITF could act as a target-gene for miR-585-5p. Interestingly, we carefully examined all predicted target-genes and found that among them, two have been reported to be associated with MITF: CREB1 and MAPK1. CREB1 has been widely confirmed to bind to a site of the MITF promotor, upregulating its expression (15). Phosphorylation of MITF by MAPK1 at serine 73 enhances its transcriptional activity (16). To further ascertain promising binding sites, we employed alignments for miR-585-5p seed sequence and 3’ UTR sequences of MITF, CREB1 and MAPK1, combined with another methodology of free energy calculation (Detailed predicted information is provided in Appendix S2). The above analysis implies that MITF might be the core target by which miR-585-5p works in GC progression. Considering that the prediction of CREB1 and MAPK1 suggest the probability of MITF regulation, we sought to determine the role of MITF.

**Figure 3:**
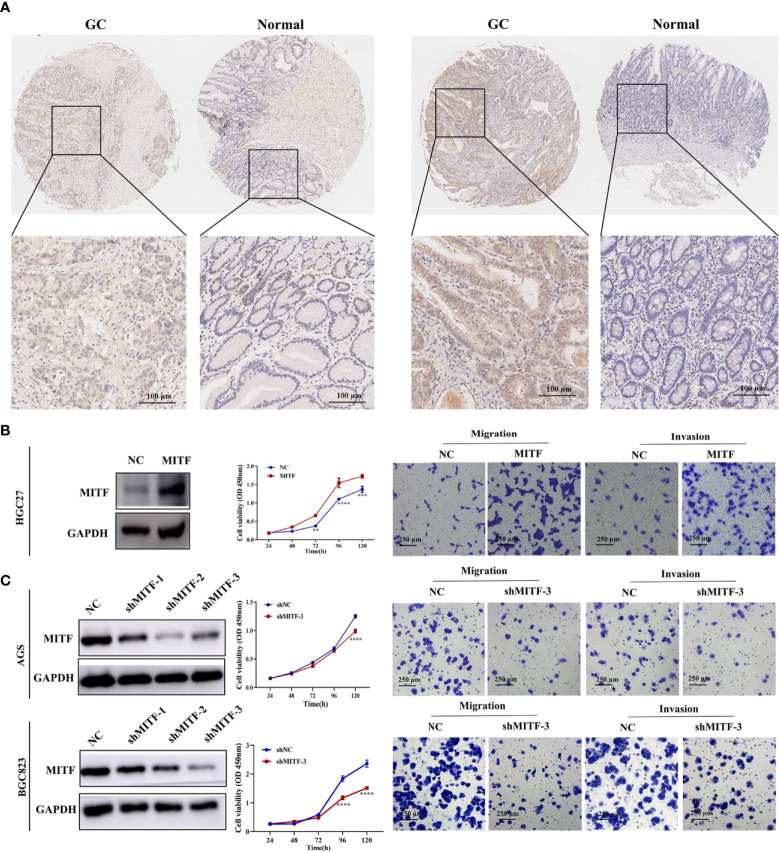
MITF is upregulated in human GC tissue-types and exacerbates GC proliferative/metastatic properties. **(**A**)** IHC staining for MITF in GC tissue-types and paired paraneoplastic tissue-types upon a series of tissue microarray samples. Representative images of MITF in GC and paired paraneoplastic tissue-types. **(B)** Western blot for detecting enforced expression of MITF in HGC27 cultures following infection with MITF lentiviruses. MITF overexpression resulted in elevated proliferation and migratory behaviours in HGC27 cultures. **(C)** Blocking efficiency of shMITF lentivirus infection in AGS and BGC823 cultures was tested by western blotting. Based on the detected efficiency, shMITF-3 lentivirus was used for follow-up functional experiments. Loss of MITF dampened proliferative/metastatic properties in AGS and BGC823 cultures, as demonstrated by CCK-8 and Transwell assays. Datasets reflected mean ± SEM. **P < 0.01, ***P < 0.001 and ****P < 0.0001 in comparison with NC cohort. Scale bar = 100 μm or scale bar = 250 μm.

**Figure 4:**
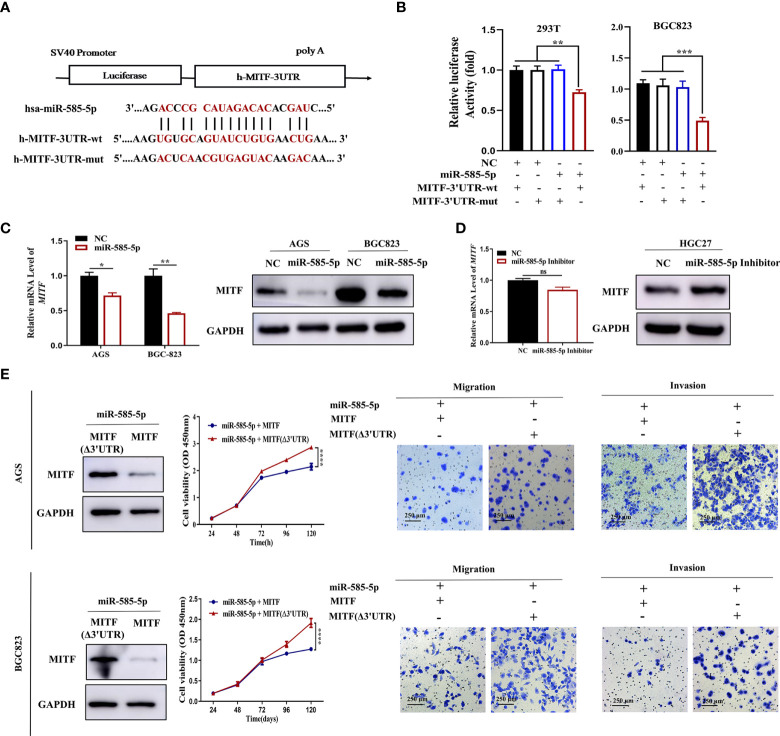
MiR-585-5p inhibits GC malignant development by negatively regulating MITF expression. **(A)** Diagram of potential binding sites for miR-585-5p within MITF 3’UTR. Mutation was generated within binding site. **(B)** Direct recognition of the MITF-3’UTR by miR-585-5p *via* luciferase reporter assay. 293T and BGC823 cultures were co-exposed to transfection with wild-type or mutant MITF fused with firefly luciferase reporters and miR-585-5p or negative control. Luciferase activity was assayed at 48 h following transfection. MiR-585-5p highly repressed luciferase function within wt-MITF luciferase reporters though not mutant reporters. **(C)** Upregulated levels of miR-585-5p led to transcriptomic/proteomic MITF downregulation. **(D)** Inhibition of miR-585-5p resulted in opposite changes in MITF protein levels but no significant change in mRNA levels. **(E)** Overexpression of MITF without miR-585-5p-binding sites. MITF (Δ3’UTR) antagonized inhibition of proliferative/metastatic phenotypes of AGS and BGC823 GC cultures driven through miR-585-5p. Datasets reflected mean ± SEM. *P < 0.05, **P< 0.01, ***P < 0.001, ****P < 0.0001 and ns suggested nil significance in comparison with NC cohort. Scale bar = 250 μm. ns indicates no significance.

Based on IHC staining, we detected MITF upregulation in GC tissue-types compared to para-cancerous tissue-types (**Figure 3A**). Validating MITF function within GC, further gain-of-function experiments indicated that MITF overexpression increases the proliferative, migrative and invasive effects of GC cultures but that downregulation has opposing effects (**Figures 3B, C**). To assess whether miR-585-5p directly inhibits MITF translation, we constructed 3’UTR reporter genes for MITF-containing miR-585-5p binding sites as reporter-genes for binding-site mutations (**Figure 4A**) and co-exposed to transfection miR-585-5p mimics into 293T and BGC823 cultures. Luciferase activity outcomes demonstrated ectopic overexpression of miR-585-5p impaired activity of the MITF-3’UTR-wt reporter, though there was no notable activity shifts within mutated reporter (**Figure 4B**). Furthermore, RT-qPCR and western blot analyses were carried out employing GC cultures to explore the regulatory MITF function by miR-585-5p. Upregulation of miR-585-5p led to transcriptomic/proteomic MITF downregulation (**Figure 4C**). Decreased levels of miR-585-5p led to dramatic proteomic MITF upregulation; however, no significant change in MITF mRNA was found within miR-585-suppressed HGC27 cultures (**Figure 4D**).

Given the crucial role of MITF in GC development, we wondered whether MITF contributes to the miR-585-mediated GC phenotype. To clarify whether miR-585-5p regulates GC cell growth and metastasis inhibition through MITF, we adopted an MITF antagonism-of-function strategy employing the MITF expression vector without miR-585-binding sites in miR-585-5p-co-expressing AGS and BGC823 cultures and found that MITF overexpression reverses the *in vitro* inhibitory effect of miR-585-5p on proliferative/metastatic properties in GC (**Figure 4E**).

### MiR-585-5p inhibits MITF transcription by directly targeting CREB1

**Figure 5:**
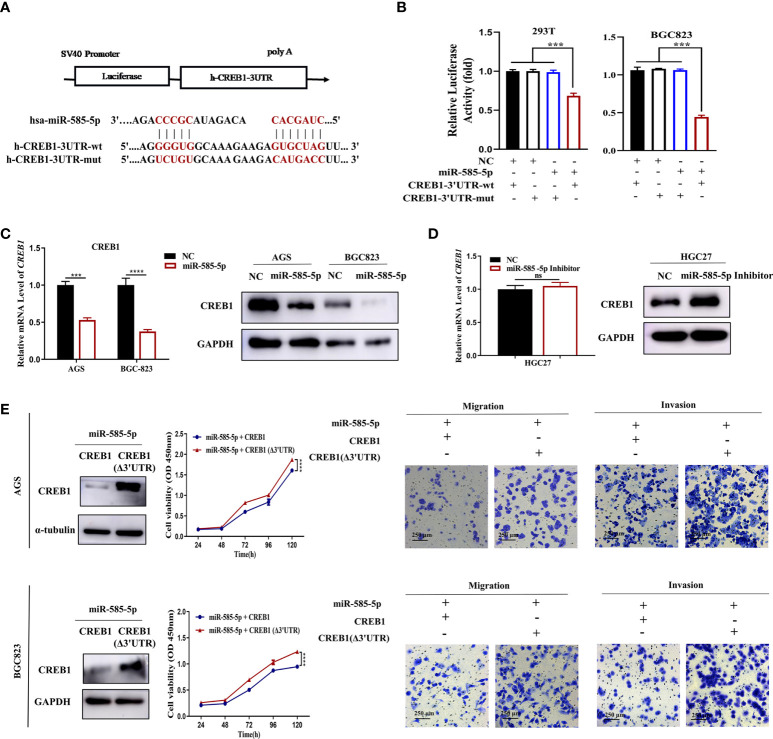
MiR-585-5p directly targets CREB1. **(A)** Potentially conserved miR-585-5p binding site within CREB1 3’UTR. Mutation was generated within binding site. **(B)** Luciferase reporter assay for miR-585-5p and CREB1. MiR-585-5p markedly impeded luciferase activities of the reporter harbouring the wt-MITF-3’UTR. **(C)** Elevated miR-585-5p resulted in CREB1 downregulation. **(D)** Inhibiting miR-585-5p led to proteomic upregulation for MITF though no significant change within mRNA level. **(E)** CREB1 overexpression reversed the anti-proliferation, anti-migrative and anti-invasive effects of miR-585-5p in AGS and BGC823 cultures. An expression vector dislodged with the miR-585-binding sites, CREB1 (Δ3’UTR), was used to enhance CREB1 expression. Datasets reflected mean ± SEM. ***P < 0.001, ****P < 0.0001 and ns indicate no significance in comparison with NC cohort. Scale bar =250 μm.

In our previous study, bioinformatic analysis demonstrated CREB1 to act as an underlying target for miR-585-5p. Multiple studies have confirmed that CREB1 directly activates transcription of the MITF gene (17). Consequently, we evaluated whether CREB1 is an imperative intermediate bridging miR-585-5p and MITF. To explore presumed miRNA-mRNA interactions between miR-585-5p and CREB1, the entire CREB1 3’UTR harboring potential miR-585 binding sites and the corresponding mutant CREB1 3’UTR were fused to a reporter vector downstream of the firefly luciferase gene (**Figure 5A**). The resulting plasmid was exposed to transfection into 293T and BGC823 cultures along with miR-585-5p mimics and a transfection negative control. As expected, miR-585-5p overexpression highly interfered with the luciferase activity of the CREB1-wt reporter, and the inhibitory effect was antagonized by transfection of the CREB1-mut reporter (**Figure 5B**). Moreover, cellular CREB1 levels were robustly reduced by introduction of miR-585-5p into AGS and BGC823 cultures (**Figure 5C**). Conversely, the use of miR-585-5p inhibitors in HGC27 cultures promoted proteomic CREB1 augmentation though not at transcriptomic level (**Figure 5D**). Taken together, such results show that CREB1 is directly targeted by miR-585-5p.

To determine whether the suppressive effects on GC phenotypes exerted by miR-585-5p are related to CREB1, we first specified the role of CREB1 in GC. CREB1 overexpression lentivirus was used to infect HGC27 cultures, and exogenous CREB1 overexpression potently stimulated cell proliferative, migrative and invasive effects *in vitro* (**Supplementary Figure 2A**). In contrast, CREB1 knockdown in AGS and BGC823 cultures employing shRNA lentiviruses exhibited opposite effects on GC phenotypes (**Supplementary Figure 2B**). In order to increase clarity on the functional links across miR-585-5p/CREB1, we applied a CREB1 gain-of-function approach in miR-585-expressing AGS and BGC823 cultures, and found that CREB1 (Δ3’UTR) upregulation partly rescued inhibitory effects of miR-585-5p on GC cell proliferative/metastatic properties (**Figure 5E**). Collectively, Such dataset outcomes suggest that miR-585-5p might regulate proliferative/metastatic properties in a CREB1-dependent manner.

**Figure 6:**
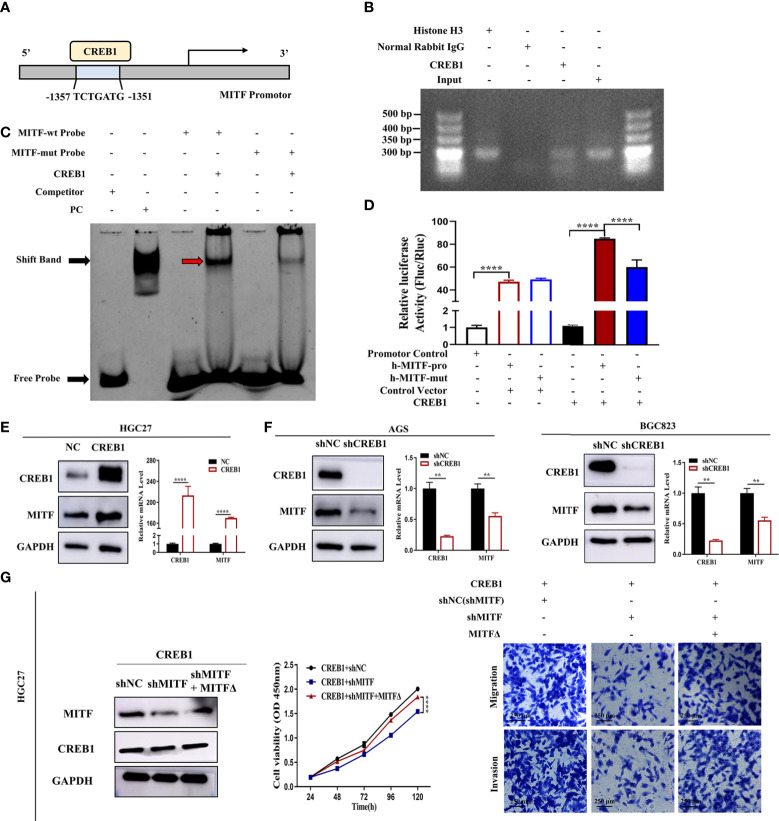
CREB1 positively enhances MITF transcription. **(A)** Graphical representation for MITF promotor. Blue region indicates predicted CREB1-binding site. Mutations were created within binding site. **(B)** Amplification of binding area for MITF promoter from ChIP DNA in HGC27 cultures. Histone H3 and normal rabbit IgG acted as positive and negative control, respectively. Nucleic acid electrophoresis for PCR products highlighted CREB1 protein binds directly onto MITF promotor at the indicated sites. **(C)** EMSA for identification of CREB1-binding sites within MITF promotor. The purified recombinant CREB1 protein was induced and obtained from *Escherichia coli* BL21. CREB1 was incubated with a biotin-labelled DNA probe, followed by chemiluminescent EMSA. Mutant probe acted as a negative control and the unlabelled probe as cold competitor. Red arrow indicates CREB1 DNA-binding complex. The use of the MITF-mut probe hindered the DNA-protein (CREB1) complex band shift. **(D)** Reporter gene analyses for transcriptional activation capacity by MITF promoter. Constructs with an intact or mutant MITF promoter resulted in enhanced luciferase activity in CREB1-overexpressing 293T cultures. The MITF-mut promoter highly suppressed luciferase activity in contrast with the MITF-wt promoter, indicating that the region from -1357 to -1351 bp is a CREB1-responsive region but not the only one. **(E)** MITF mRNA and protein levels in CREB1-overexpressing HGC27 cultures. CREB1 overexpression remarkably promoted MITF expression. **(F)** MITF mRNA and protein levels in CREB1-knockdown AGS and BGC823 cultures. Downregulated CREB1 inhibited MITF expression. **(G)** Downregulated MITF expression in HGC27-CREB1 cultures blocked cell proliferative, migrative and invasive effects driven through CREB1 overexpression. An shMITF-resistant reconstitution, MITFΔ, was employed for rescuing MITF expression, recovering increased malignant ability of cultures. Datasets reflected mean ± SEM. **P < 0.01, ***P < 0.001 and ****P < 0.0001 in comparison with NC cohort. Scale bar = 250 μm.

As an important transcription factor, CREB1 binds to the cAMP response element (CRE) consensus motif situated across -140 and -147 bp from the transcription site of the MITF promoter to enhance its expression (15). Multiple studies have affirmed the positive regulation of MITF transcription by CREB1 in malignant melanoma (18–20). However, whether CREB1 directly activates MITF transcription in GC is still unclear. Therefore, we predicted the interactive mode of the CREB1 protein and MITF promotor region employing the JASPAR database (http://jaspar.genereg.net/), and the highest scoring CREB1 binding site, i.e., TCTGATG (-1357 to -1351), was selected *via* bioinformatics analysis (**Figure 6A**). To confirm the hypothesis that MITF is a key target-gene of CREB1 in GC, ChIP-PCR was performed, and nucleic acid electrophoresis analysis showed that the CREB1 protein bound directly to the MITF promotor at the indicated sites in HGC27 cultures (**Figure 6B**). Subsequently, a probe for this binding site was designed and synthesized, and EMSA was carried out. Such dataset outcomes demonstrated proteomic CREB1 strongly binds to the MITF-wt probe; however, the interaction was highly but not completely hindered, which further confirms that CREB1 might bind to this site but that it is not the only binding site (**Figure 6C**). Additionally, the CREB1 expression vector was co-exposed to transfection with the MITF full-length promoter reporter gene or the binding-site mutant reporter gene into 293T cultures. CREB1 highly upregulated the transcriptional activity of the MITF wild-type promoter; the regulatory effect was inhibited but still existed within presence of the -1357 to -1351 binding site mutation, suggesting that CREB1 regulates MITF transcriptional expression through this binding site but not the only binding site (**Figure 6D**).

A CREB1 overexpression lentivirus was used to infect HGC27 cultures, and shCREB1 lentivirus was applied to knockdown CREB1 in AGS and BGC823 cultures. Upregulated CREB1 obviously upregulated transcriptomic/proteomic MITF expression (**Figure 6E**), whereas downregulating CREB1 resulted in dramatically decreased MITF expression (**Figure 6F**). Based on the finding that CREB1 accelerates proliferative/metastatic properties in GC cultures, this investigation probed the possibility whether CREB1 functions in an MITF-dependent manner. Hence, we infected CREB1-overexpressing HGC27 cultures with shMITF lentivirus or shNC lentivirus and carried out CCK-8 and Transwell assays. Overall, knockdown of MITF diminished the CREB1-mediated promotion of cell growth and metastasis in GC (**Figure 6G**). In addition, upregulation of MITF rescued the dampened tumour proliferative/metastatic properties driven by shRNA-mediated silencing of CREB1 (**Supplementary Figure 3**). These findings suggest that CREB1-induced MITF overexpression promotes GC proliferative/metastatic properties. In summary, such dataset outcomes verify that miR-585-5p inhibits MITF transcription through direct targeting of CREB1.

### MiR-585-5p suppresses MITF activity by directly targeting MAPK1

**Figure 7:**
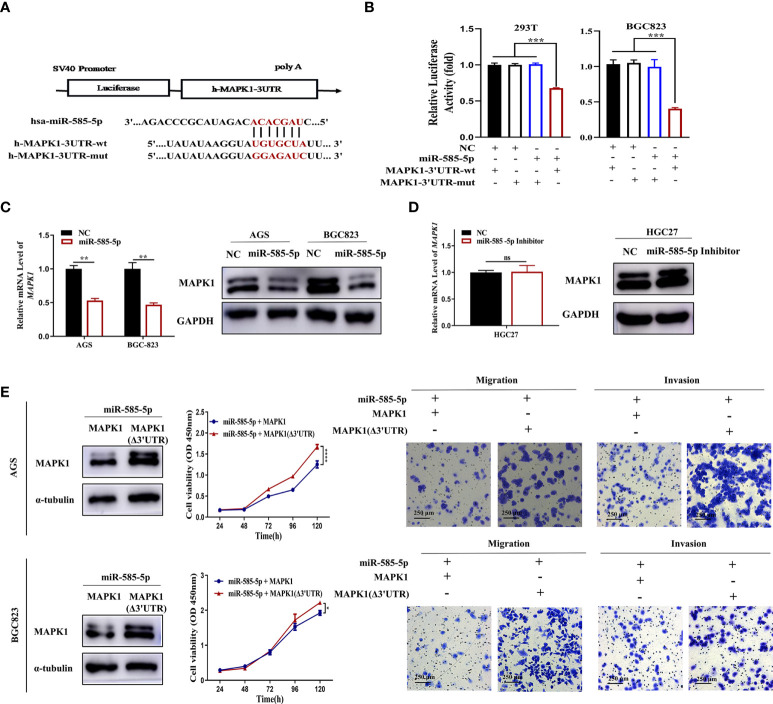
MiR-585-5p directly targets MAPK1. **(A)** Potentially conserved miR-585-5p binding site within MAPK1 3’UTR. Mutation was generated within binding site. **(B)** Verification of MAPK1 as a miR-585-5p target *via* luciferase reporter assays. MiR-585-5p highly inhibited the luciferase activities for reporter harbouring the wt-MITF-3’UTR though not mut-MITF-3’UTR. **(C)** Elevated miR-585-5p resulted in MAPK1 transcriptomic/proteomic downregulation. **(D)** Inhibiting miR-585-5p drove proteomic MAPK1 upregulation, though no significant change in mRNA. **(E)** MAPK1 (Δ3’UTR) overexpression reversed the anti-proliferative, anti-migrative and anti-invasive influence by miR-585-5p in AGS and BGC823 cultures. Datasets reflected mean ± SEM. *P < 0.05, **P < 0.01, ***P < 0.001, ****P < 0.0001 and ns indicates no significance in comparison with NC cohort. Scale bar =250 μm.

Based on the previous prediction of MAPK1 as a target-gene of miR-585-5p, this investigation examined if miR-585-5p is able to regulate MAPK1 in GC. The luciferase reporter system validated that miR-585-5p could be directly-bound onto MAPK1-3’UTR at the indicated sites (**Figures 7A, B**). Moreover, the transcriptomic/proteomic MAPK1 downregulation occurred by ectopic expression of miR-585-5p in AGS and BGC823 cultures (**Figure 7C**). Consistently, miR-585-5p inhibitors led to elevated expression of MAPK1 (**Figure 7D**). It is widely recognized that MAPK1 plays has pivotal parts within development and progression of various cancers, including GC (21–23). However, whether MAPK1 is implicated within miR-585-mediated tumour proliferative/metastatic properties remains uncertain. Resistance-of-function experiments were conducted *via* overexpression of MAPK1 with or without miR-585-5p-binding sites, showing that MAPK1 reverses the anti-proliferative, anti-migrative and anti-invasive capacities of miR-585-5p in AGS and BGC823 GC cultures (**Figure 7E**). Overall, dataset outcomes suggest MAPK1 is a functional target of miR-585-5p within GC malignant phenotype.

**Figure 8:**
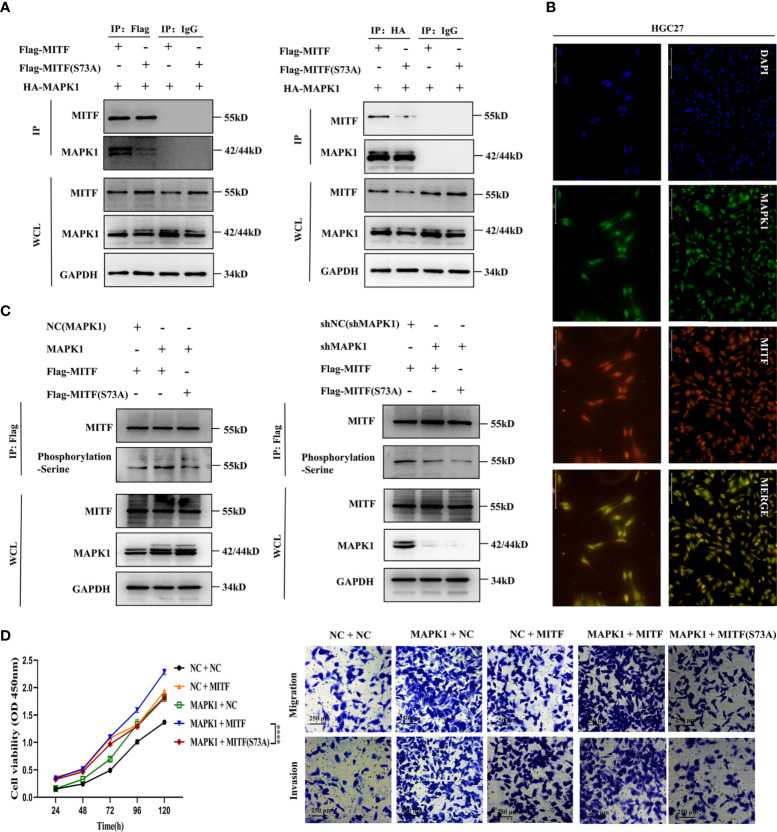
MAPK1 enhances the cancer-promoting characteristics of the MITF protein *via* phosphorylation at serine 73. **(A)** Endogenous immunoprecipitation was performed to verify interaction between MAPK1 and the MITF protein or the MITF(S73A) protein in HGC27 cultures. MAPK1 and MITF or MAPK1 and MITF (S73A) were co-expressed in HGC27 cultures. When the MITF protein was immunoprecipitated by an anti-flag antibody, the indicated proteins were detected by immunoblotting. The same was true for immunoprecipitation of the MAPK1 protein *via* an anti-HA antibody. Normal rabbit IgG acted as negative control. **(B)** Intracellular colocalization between MAPK1 and MITF was observed by immunofluorescence staining of parental HGC27 cultures. **(C)** MAPK1 phosphorylated the MITF protein at serine73. The MITF protein was immunoprecipitated by an anti-flag antibody employing NC(MAPK1)+MITF, MAPK1+MITF and MAPK1+MITF(S73A) coexpressing HGC27 cultures. Serine phosphorylation levels were detected by immunoblot employing an anti-phosphorylation (serine) antibody. Upregulated MAPK1 enhanced serine phosphorylation of MITF protein, whereas MAPK1 knockdown restrained this effect. Mutation of serine 73 of the MITF protein antagonized MAPK1-mediated phosphorylation. **(D)** CCK-8, Transwell migration and invasion assays within indicated cultures. MAPK1 overexpression notably enhanced the proliferation-promoting and metastasis-promoting effects of MITF but not mutant MITF (S73A). Datasets reflected mean ± SEM. ****P < 0.0001 in comparison with NC cohort. Scale bar = 75 μm, scale bar = 125 μm or scale bar = 250 μm.

The transcriptional and MITF functional activity is founded upon post-translational modifications and the availability of cooperating partners. MAPK1 was confirmed to phosphorylate the MITF protein at serine 73, enhancing its activity (16, 24). However, whether this effect exists in GC is indeterminate. To assess the interplay between MAPK1 and MITF, we overexpressed HA-tagged MAPK1 and flag-tagged MITF mutants (S73A) or wild-type MITF at similar levels in HGC27 cultures and used immunoprecipitation to evaluate the interaction. The MAPK1-MITF interaction was confirmed by blotting complexes containing endogenous MITF precipitated with flag-specific antibodies and probing for MAPK1. Conversely, an anti-HA antibody was used for precipitation, and the isolated complexes were blotted with an anti-MITF antibody. The interaction was partly abolished when the S73A mutant protein was used (**Figure 8A**), and an endogenous interaction assay studying GC cultures demonstrated that MAPK1 interacts with MITF and that serine 73 is a crucial site. Furthermore, immunofluorescence was employed to determine the cellular distributions of the two proteins, and the superimposition of green fluorescence indicating MAPK1 over red fluorescence indicating MITF validated the association between MAPK1 and MITF (**Figure 8B**).

Interestingly, we found that MAPK1 overexpression or silencing had no significant influence on MITF levels at either the mRNA or protein level (**Supplementary Figures 4A, B**), suggesting that MAPK1 might only be implicated within phosphorylation of MITF but not in its degradation. To assess MAPK1 phosphorylation of the MITF protein at serine 73, we conducted combinatorial expression of NC(MAPK1) + MITF, MAPK1 + MITF and MAPK1 + MITF(S73A) in HGC27 cultures. Phosphorylated MITF was analysed by employing an anti-phosphorylation (serine) antibody to detect the immunoprecipitated MITF protein, showing that overexpressing MAPK1 strongly increased global MITF phosphorylation in GC cultures but that overexpressing the S73A mutant did not. In contrast, MAPK1 silencing attenuated the phosphorylation level of MITF, and S73A mutants sustained low-level phosphorylation, suggesting that serine 73 is a principle site for phosphorylation (**Figure 8C**). Collectively, the above results reveal that MAPK1 binds to MITF and phosphorylates serine 73 in GC cultures. In order to increase clarity regarding MAPK1 function in MITF serine phosphorylation, HGC27 cultures were engineered to express elevated levels of MAPK1 and MITF or mutant MITF (S73A) alone or in combination. We also overexpressed wild-type MITF or the S73A mutant in HGC27 cultures with downregulated MAPK1 to comprehensively assess the functional effect of MAPK1-mediated phosphorylation on MITF serine73. CCK-8 and Transwell assays showed that compared with NC + NC cultures, MAPK1 or MITF alone enhanced proliferative/metastatic properties, and the MAPK1 + MITF co-expressing cultures exhibiting the most strongly enhanced malignant phenotypes. However, MAPK1+MITF (S73A)-overexpressing cultures presented highly dampened proliferative and metastatic capacities relative to MAPK1 + MITF co-expressing cultures, which indicated that MAPK1 overexpression notably enhances the cancer-promoting characteristics of MITF but not of the MITF (S73A) mutant (**Figure 8D**). Taken together, our findings indicate that MAPK1 enhances the cancer-promoting characteristics of the MITF protein *via* serine 73 phosphorylation. In summary, results indicated miR-585-5p suppresses MITF activity by directly targeting MAPK1.

## Discussion

**Figure 9:**
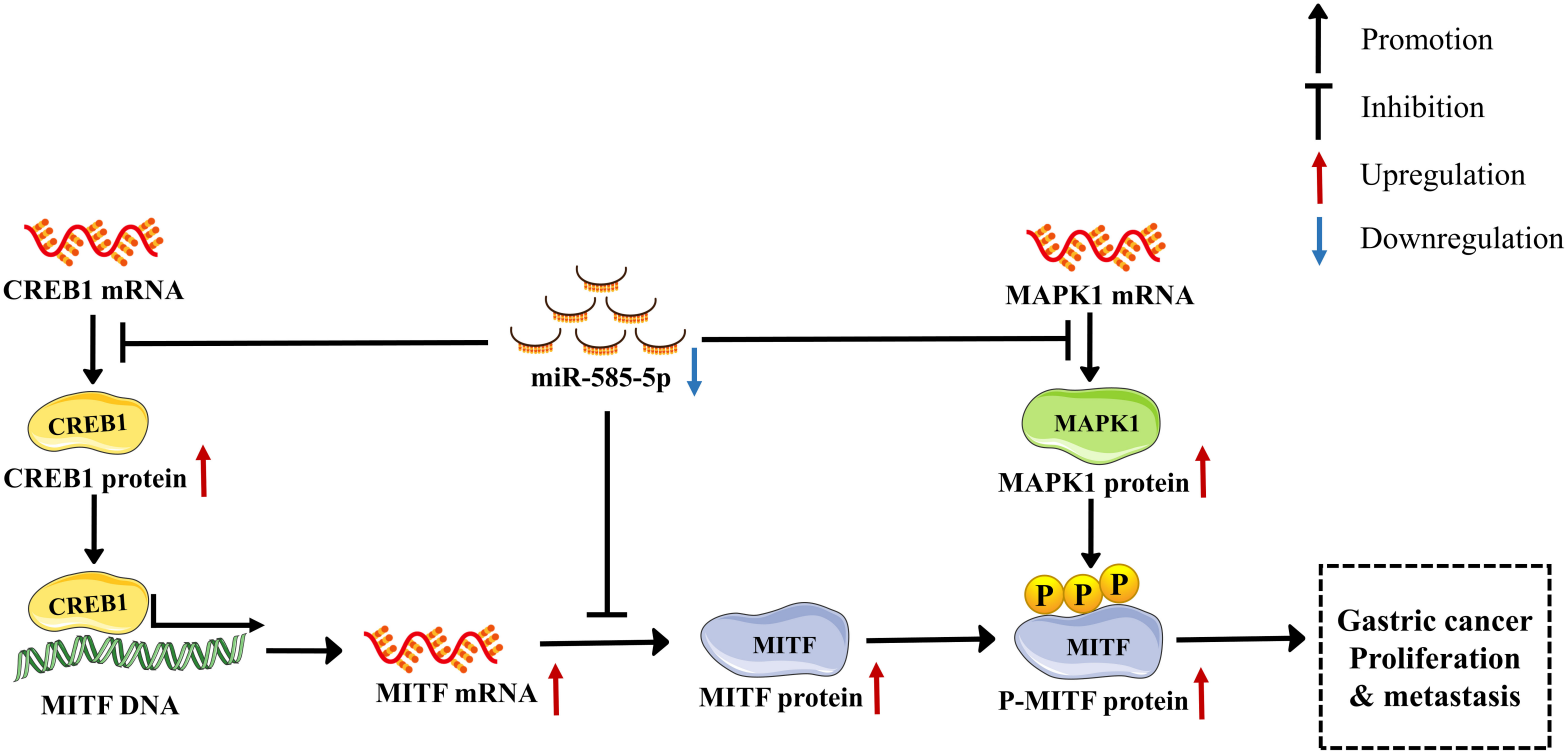
A new miR-585-5p/(CREB1/MAPK1/MITF) signalling pathway inhibits GC (GC) proliferative/metastatic properties. MiR-585-5p was downregulated in GC development, resulting in increased CREB1, MAPK1 and MITF expression. Expression of CREB1, MAPK1 and MITF was upregulated. In particular, upregulated CREB1 activated MITF transcription, further promoting MITF upregulation. MAPK1 phosphorylates the MITF protein at serine 73, which activates MITF pro-cancerous activity, promoting GC proliferative/metastatic properties.

This investigation revealed miR-585-5p is markedly downregulated in GC tissue-types and that cases of positive miR-585-5p expression have better clinical outcomes than cases of negative miR-585-5p expression. Furthermore, we identified for the first time the melanocyte master regulator MITF as promoting carcinogenesis in GC and acting as a direct and essential mediator of miR-585-5p-impeded malignant phenotypes. Such dataset outcomes demonstrated that overexpression of miR-585-5p highly inhibits proliferative/metastatic properties of GC by directly targeting MITF, CREB1 and MAPK1. Moreover, CREB1 directly regulates MITF transcription, and MAPK1 directly phosphorylates MITF at serine73. This interaction network shows that miR-585-5p directly or indirectly regulates MITF expression and activity by simultaneously repressing target molecules, accounting for the anti-tumour role of miR-585-5p in inhibiting GC growth and metastasis. In summary, our study supports that miR-585-5p suppressed GC proliferative/metastatic properties through CREB1/MAPK1/MITF pathway (**Figure 9**).

MiRNAs, an abundant class of endogenous noncoding RNAs, have been confirmed to be ectopically expressed in malignant gastric tissue-types. Dysregulated miRNAs are important in regulating proliferative/metastatic properties of GC (25, 26). The gene encoding miR-585-5p is located at 5q35.1 within intron of SLIT3, together with miR-218-5p (6). Notably, miR-218-5p was previously identified as a tumour suppressor in GC by our research cohort (4, 5), and we tentatively proposed that miR-585-5p has a similar expression pattern and phenotypic effects as miR-218-5p in GC, considering that the two miRNAs belong to the same gene cluster. MiR-585 has been previously characterized to be a tumour suppressor in non-small-cell lung cancer (9), triple-negative breast cancer (27), cervical cancer (28), glioma (10), tongue squamous cell carcinoma (29), and colorectal cancer (30), among others. However, few studies have determined the specific role of miR-585 in GC. Some reports have identified that miR-585 exhibits relatively low expression in GC tissue-types, predicting poor prognosis (11, 31), but the molecular mechanisms implicated are far from clear. Several studies report that miR-218 directly targets MITF to exert its biological functions (32–34), and we accordingly screened for potential targets of miR-218-5p and miR-585-5p; MITF was co-pinpointed by bioinformatic prediction algorithms. However, inconsistent with existing studies, our findings showed that miR-218-5p overexpression had no impact on MITF expression (**Supplementary Figure 5**) but that miR-585-5p was affirmed to directly target and regulate MITF in a post-transcriptional manner. Herein, we focus on miR-585-5p implications and its downstream molecular mechanisms in GC. We not only reconfirmed decreased expression of miR-585 in GC but, more importantly, highlighted that miR-585-5p hinders GC proliferative/metastatic properties by directly or indirectly adjusting MITF expression and biological activities at different levels of gene expression regulation. In addition, we identified that miR-585-5p simultaneously targets CREB1 and MAPK1 mRNAs, downregulating transcriptomic/proteomic expression. Functionally, miR-585-5p overexpression inhibits GC cell proliferative, invasive, and migrative properties by targeting CREB1 and MAPK1 in resistance-of-function analyses. In agreement with our results, Hu et al. (11) reported that miR-585 is downregulated in both GC tissue-types and cultures and that ectopic overexpression highly suppresses the malignant phenotype of GC by directly targeting MAPK1, with no evidence that miR-585-5p directly regulates CREB1. Additionally, miR-585 binds to the 3’UTR of F-box protein 11 (FBOX11), and overexpression of miR-585 inhibits the GC cell proliferation and migration driven through FBXO11 (31). Taken together, our findings indicate that miR-585-5p expression is often decreased in GC, which is of great value in GC treatment *via* suppression of tumour growth and metastasis. Such dataset outcomes illustrate for the first time that the anti-tumour effect of miR-585-5p in GC is due to direct simultaneous inhibition of MITF, CREB1 and MAPK1 expression.

MITF, widely identified as one of the most classic and pivotal regulators in malignant melanoma, has been associated with phenotypic switching between predominantly invasive and proliferative behaviours of melanoma. Notably, MITF has been reported as a tumour suppressor in inhibiting tumour growth and metastasis in GC (35, 36). Several bodies of proof partly support that MITF plays an oncogenic function within GC development. For instance, miR-876-5p suppresses GC cell viability/migrative properties though induces apoptosis by targeting MITF (36). Li et al. (35) inferred that CSE1L silencing promotes apoptosis and inhibits tumour growth and metastasis by decreasing MITF expression. Nevertheless, whether MITF is aberrantly expressed and has a pathogenic function within GC, remains largely elusive. This investigation detected MITF expression in GC tissue-types for the first time, revealing that MITF was upregulated in GC tissue-types in comparison with peri-carcinomatous tissue-types. Within subsequent gain- and loss-of-function analyses, we observed that MITF overexpression conspicuously promotes HGC27 cell proliferation, invasion and migration but that MITF downregulation highly suppresses the malignant phenotypes of AGS and BGC823 cultures. Hence, in contrast to the controversial context-dependent regulation of MITF within tumorigenesis and progression of melanoma, our findings show that MITF acts straightforwardly as an oncogene in GC. In terms of the underlying mechanisms driving *de novo* MITF expression in GC, MITF was proven to be a direct target of miR-585-5p. Such dataset outcomes suggest that the high ectopic expression of the MITF protein in GC tissue-types might be due, at least in part, to inhibitory regulation by miR-585-5p. Collectively, we report that MITF is not only a potent marker predicting prognosis but as a direct downstream target of miR-585-5p, also a pro-proliferative and pro-metastatic gene in GC.

Although the direct post-transcriptional miRNA-mRNA regulatory interaction matters greatly in regulating MITF protein expression, many transcription factors govern MITF transcriptional expression (17). As previously illustrated, we have shown that CREB1 and MITF are both the downstream targets of miR-585-5p, and it has been widely demonstrated that CREB1, a canonical bZIP transcription factor, identifies the CRE motif ‘-TGACGTCA-’ within MITF promotor (15), thus enabling varied cAMP levels to influence MITF expression (37, 38). This idea is further perpetuated by various studies showing that CREB1 directly upregulates MITF expression in human melanocytes or melanoma cultures (39–41). Nevertheless, there is a paucity of evidence regarding whether CREB1 directly activates MITF transcription in GC. This investigation is the first to confirm in GC cultures that CREB1 directly binds to the newly identified MITF promotor sites -1357 to -1351 bp, positively regulating MITF transcription. As expected, MITF mRNA and protein levels increase in response to CREB1 overexpression, whereas CREB1 knockdown results in highly decreased MITF expression. Further resistance of function showed that the deficiency of MITF in CREB1-overexpressing HGC27 cultures counteracted the pro-proliferation and pro-metastasis effects due to CREB1, underscoring that CREB1-dependent MITF upregulation is crucial to stimulate GC development. Interestingly, our results showed that the -TCTGATG- (-1357 to -1351) site might not be the only binding site for CREB1 within MITF promotor, and there is a possibility that other sites also contribute to CREB1 binding. Considering the widely confirmed CREB1 binding site TGACGTCA (-140 to -147 bp) within MITF promotor, we speculate that the two candidate sequences might both act immensely within transcriptional regulation of MITF. However, this should be further explored in GC cultures. Together with our data from ChIP, EMSA and luciferase reporter assays, we determined that CREB1 positively regulates MITF transcription in GC.

In addition to transcriptional and post-transcriptional regulation of MITF expression, regulation of MITF activity contributes to its function in tumours. Phosphorylation is a prerequisite modification for modulating MITF activity, and the MITF protein has been reported to be phosphorylated by MAPK1 at Ser73, by P90RSK at Ser409, by GSK3 at Ser69, Ser298, Ser397, Ser401, and Ser405, and by P38MAPK at Ser307. Hemesath et al. (42) first reported that MAPK1-mediated MITF phosphorylation at Ser73 enhances MITF-dependent transactivation in melanocytes, and Ser73 phosphorylation by MAPK1 is proposed to be required for recruitment of the P300/CBP transcriptional coactivator within transactivation domain of MITF (16). Phosphorylation at MITF Ser73 is predominantly responsible for MITF activation to promote malignant phenotypes in melanoma (24, 43); however, this interaction has not been reported in GC. Hence, we conducted IP and double-labelling immunofluorescence of HGC27 cultures, showing endogenous interaction between MAPK1 and MITF protein in GC, and the effect of MAPK1 phosphorylation on MITF was determined employing anti-phosphoserine antibodies. Consistent with previous studies, the S73A mutant counteracted the above effects, indicating that the serine 73 site is required for MAPK1-mediated phosphorylation of the MITF protein. The results of subsequent functional assays demonstrated that phosphorylation of serine 73 is important to facilitate MITF-enhanced GC proliferative/metastatic properties. Nonetheless, our results show that MAPK1 does not affect MITF protein levels, which is incompatible with the proposal that S73-phosphorylated MITF is a prerequisite for MITF degradation. As an E2 SUMO conjugating enzyme, UBC9 was found to be responsible for the degradation of MITF in response to S73 phosphorylation (44). Moreover, Azam et al. (45) reported that sargaquinoic acid increases phosphorylation of MAPK1 and MITF (Serine73), ultimately inducing proteasomal degradation of MITF, in melanoma cultures. Controversially, both Hemesath et al. (42) and Wellbrock et al. (46) found that the S73A mutation has no effect on MITF protein stability, in line with our results. Considering the tumour heterogeneity between melanoma and GC, together with our results, it is reasonable to speculate that MAPK1 promotes GC proliferative/metastatic properties *via* phosphorylation of MITF (Serine73), enhancing its activity instead of stability.

Additionally, accumulating lines of evidence have shown that kinase-regulated CREB1 phosphorylation activates CREB1-dependent transcription and acts as an essential cascade in oncobiology (47). Leduc et al. reported that ERK1 instead of ERK2 was necessarily required for CREB1 phosphorylation and activation in mouse pancreatic beta cells (48). Chen et al. illustrated that MAPK1 could activate CREB1 to bind to the promotor of miR-212-3p. But no specific evidence have certificated the direct interaction between CREB1 and MAPK1. Our study demonstrated that CREB1 and MAPK1 act through different approaches to promote MITF-mediated GC progression, with one enhancing MITF transcriptional expression and the other promoting phosphorylation of MITF proteins. However, our study did not address the possible regulation of CREB1 by MAPK1 in GC. Therefore, detailed analysis regarding the relationship between MAPK1 and CREB1 is needed for further investigation in GC. Besides, although the direct regulation of miR-585-5p targeting on MITF, CREB1 and MAPK1 were confirmed, miR-585-5p inhibitor did not result in significant change within mRNA levels. The results reflected that the inhibition of miR-585-5p might only make a difference to translational process instead of the stability or degradation of mRNAs of these 3 targets. And further exploration is desired for the seemingly contradictory results.

Overall, this investigation not only revealed the tumour-inhibiting role of miR-585-5p in GC proliferative/metastatic properties but also defined the mechanism of miR-585-5p downstream signalling through MITF regulation in multiple aspects of gene expression. We identified another two targets of miR-585-5p that regulate MITF: CREB1 activates MITF transcription upregulating MITF expression, and MAPK1 phosphorylates MITF (Ser73) activating MITF pro-cancerous activity. Ultimately, miR-585-5p suppresses GC development by simultaneously targeting MITF in both direct post-transcriptional and indirect transcriptional (CREB1/MITF) or post-translational (MAPK1/MITF) manners. Accordingly, miR-585-5p/MITF-based targeted therapy might be a promising strategy for cases of GC.

In conclusion, this study uncovered miR-585-5p suppressed GC proliferative/metastatic properties by orchestrating the interactions among CREB1, MAPK1 and MITF. And miR-585-5p/MITF-based targeted therapy might represent a potential therapeutic strategy.

## Data availability statement

The original contributions presented in the study are included in the article/**Supplementary Material**. Further inquiries can be directed to the corresponding authors.

## Ethics statement

All procedures involving animals were carried out in accordance with the principles of ARRIVE and approved by the Ethics Committee of Air Force Medical University.

## Author contributions

YW, WW and JT designed research. YW, ML, JZ and YY performed the experiments. YY, ML, and JZ analyzed statistics. YW, JZ and ZL purchased reagents. YW and JT wrote the article. All authors read and approved the final manuscript. All authors contributed to the article and approved the submitted version.

## Funding

This work was supported by the National Natural Science Foundation of China [81773071, 81972226] and Key R&D Program of Shaanxi Province [2020ZDLSF01-05].

## Acknowledgments

The authors would like to thank Hanbio Tech (shanghai, China) for assisting us in bioinformatic analysis. The diagram of “MITF”, “CREB1” and “MAPK1” in **Figure 9** were modified from Servier Medical Art (https://smart.servier.com) under CC BY 3.0 license (https://creativecommons.org/licenses/by/3.0/).

## Conflict of interest

The authors declare that the research was conducted within absence of any commercial or financial relationships that could be construed as a potential conflict of interest.

## Publisher’s note

All claims expressed in this article are solely those of the authors and do not necessarily represent those of their affiliated organizations, or those of the publisher, the editors and the reviewers. Any product that may be evaluated in this article, or claim that may be made by its manufacturer, is not guaranteed or endorsed by the publisher.
