# Supplementary figures and images for: MiR-585-5p impedes gastric cancer proliferation and metastasis by orchestrating the interactions among CREB1, MAPK1 and MITF
Source: Front Immunol. 2022 Oct 4;13:1008195. doi: 10.3389/fimmu.2022.1008195 (PMC9576935; doi:10.3389/fimmu.2022.1008195)

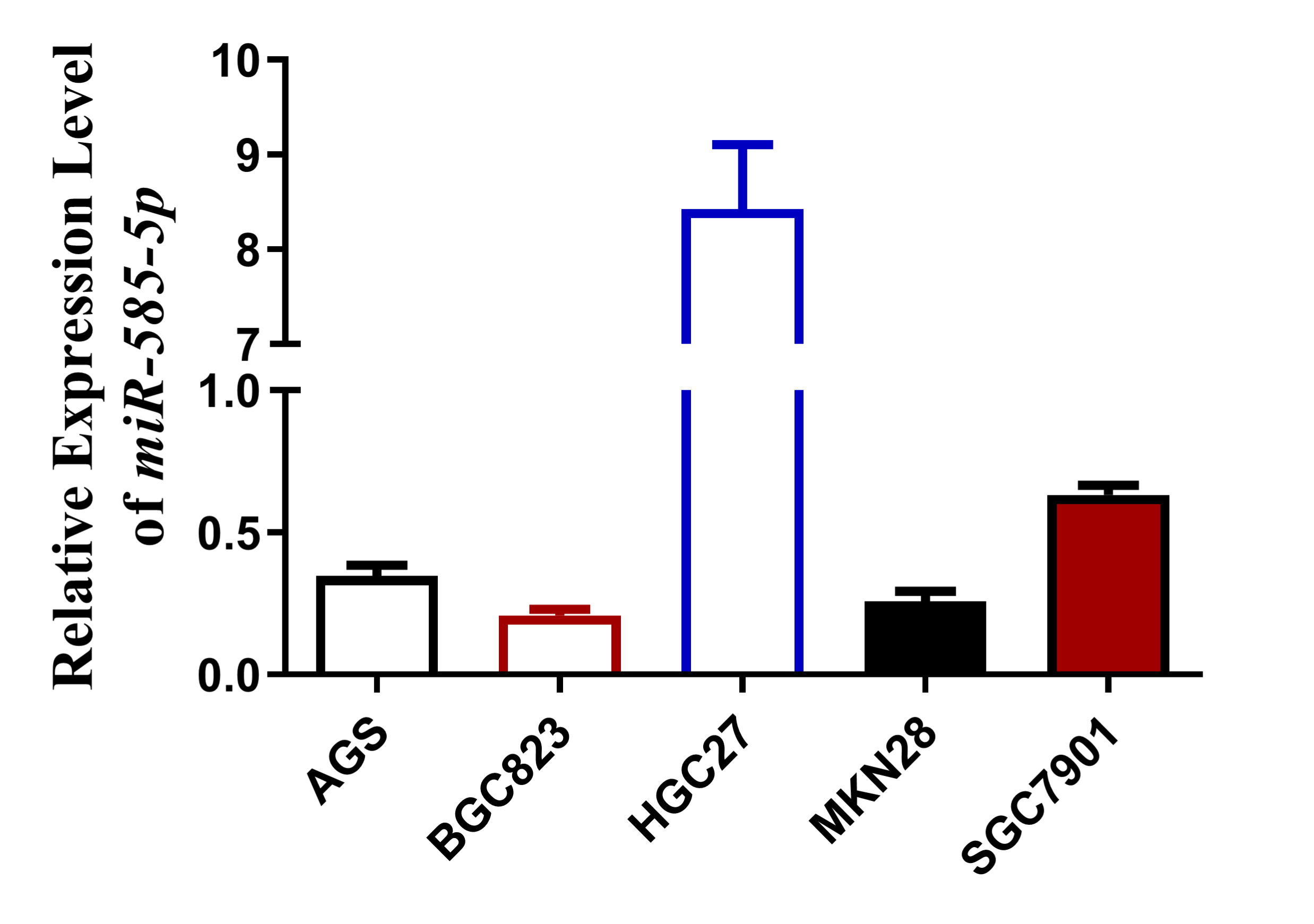

Supplement: Supplementary file 1 [file Image_1.tif]

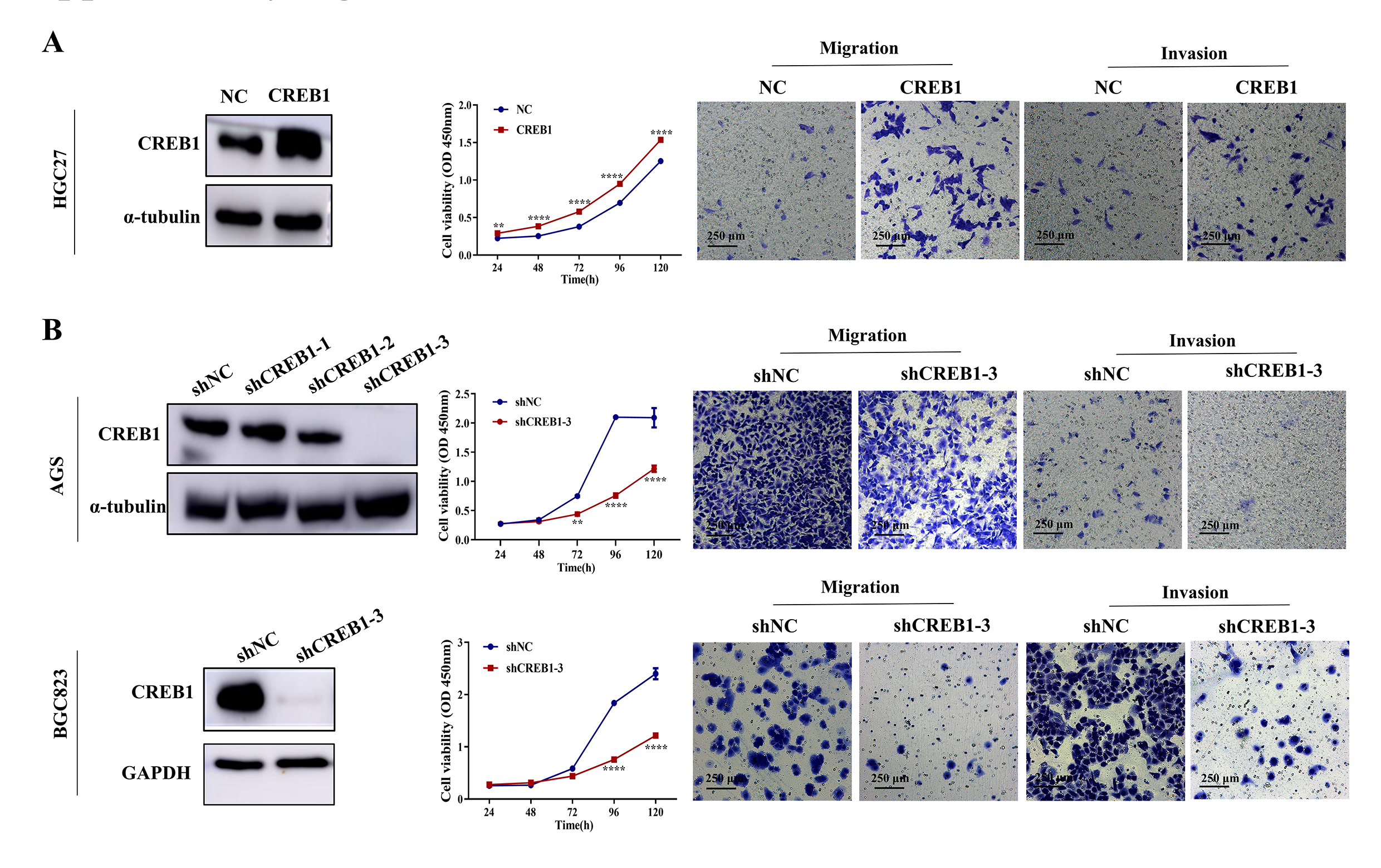

Supplement: Supplementary file 2 [file Image_2.tif]

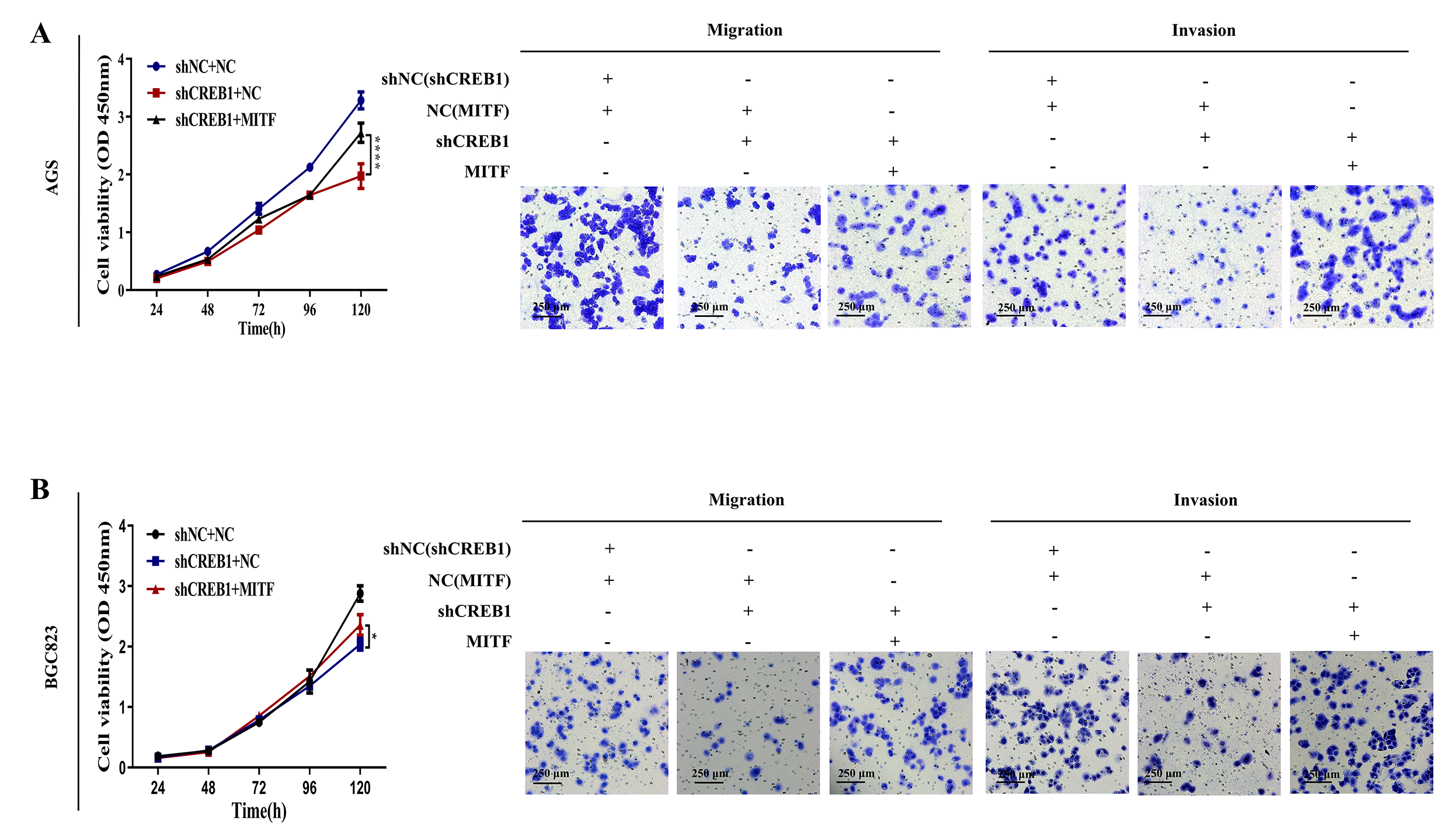

Supplement: Supplementary file 3 [file Image_3.tif]

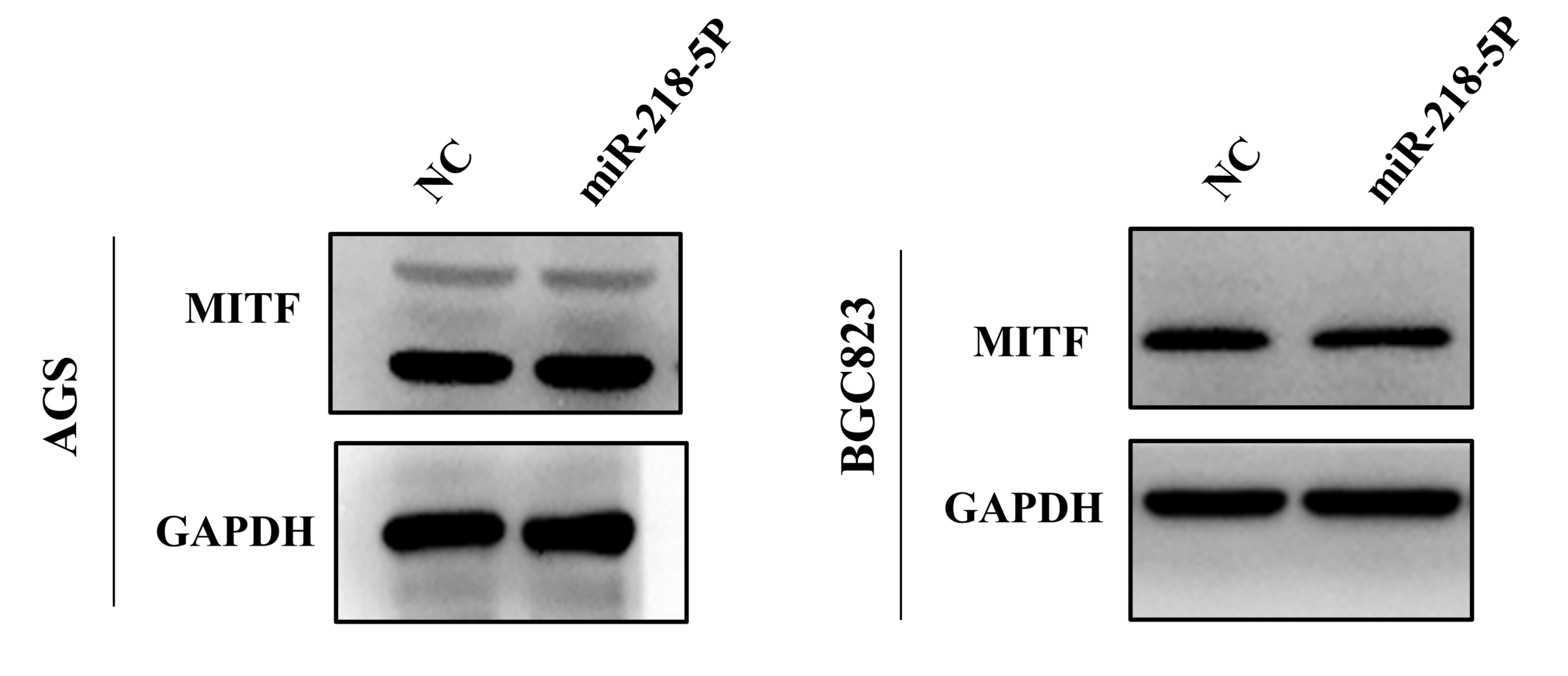

Supplement: Supplementary file 5 [file Image_5.tiff]
