# Supplementary material for: MiR-585-5p impedes gastric cancer proliferation and metastasis by orchestrating the interactions among CREB1, MAPK1 and MITF
Source: Front Immunol. 2022 Oct 4;13:1008195. doi: 10.3389/fimmu.2022.1008195 (PMC9576935; doi:10.3389/fimmu.2022.1008195)
Supplement: Supplementary file 6 [file Table_1.docx]

**Method and Materials**

**RNA isolation and qRT-PCR**

Total RNA was extracted using TRIzol reagent (Takara, #9109). In total, 1000 ng of isolated RNA was reverse transcribed into cDNA using Prime Script™ RT Reagent Kit (Takara, #RR036A-1). Quantitative PCR was performed with a CFX Connect real-time system (BIO-RAD, US) using UltraSYBR Mixture (CWBIO, #CW0957 M) and specific primers. The amplification conditions were 95°C and 10 min for pre-denaturation, followed by 40 cycles of 95°C for 10 sec and 60°C for 30 sec. Relative expression was calculated by the 2-ΔΔCt method and normalized to the GAPDH expression level. MiR-585-5p cDNA was synthesized using a miR-X miRNA First-Strand Synthesis Kit (Takara, #638315). Polyadenylation and reverse transcription were performed at 37°C for 10 sec and 60°C for 30 sec. Quantification of miRNA by qPCR was executed using SYBR Advantage qPCR Premix (Takara, #639676) with an initial denaturation step at 95°C for 10 sec, followed by 42 cycles at 95°C for 5 sec and 60°C for 20 sec. U6 snRNA was used as the internal control to normalize miR-585-5p expression. The primer sequences for each gene are listed in Table 1.

**Western blotting**

Total protein extracts were lysed in potent cell lysis buffer containing 1% protease inhibitor and boiled with loading buffer at 100°C for 10 min. Equal amounts of protein (20-40 µg) were separated via SDS-PAGE and transferred onto a polyvinylidene difluoride (PVDF) membrane (Millipore, #IPVH00010). The membranes were blocked in 5% nonfat milk dissolved in TBST for 3 h and then incubated overnight at 4°C with primary antibodies. The next day, the membranes were probed with an HRP-conjugated secondary antibody for 2 h and visualized with chemiluminescence (Millipore, #WBKLS0500) using a FluorChem FC system (Alpha Innotech). The antibodies used in this study were as follows: anti-MITF (Santa Cruz Biotechnology, # sc-56725), anti-CREB1 (Cell Signalling Technology, #9197), anti-GAPDH (Bioss, #bs-2188R), anti-MAPK1 (Cell Signalling Technology, #4695), anti-FLAG (Cell Signalling Technology, #14793), and anti-HA (Cell Signalling Technology, #3724).

**Luciferase reporter assay**

The 3’ UTRs of MITF, CREB1 and MAPK1 containing a seed region for miR-585-5p, as well as all mutant sequences, were amplified from genomic DNA by PCR. The 3’UTR fragment was amplified and cloned into the pSI-Check2 luciferase vector. HEK293T and BGC823 cells were seeded in a 96-well plate at a density of 50%-70%. On the following day, co-transfection of wild-type 3’UTR or mutant 3’UTR reporter combined with miR-585-5p mimics or NC was carried out using Lipofectamine 3000 reagent (Invitrogen, #L3000015). Luciferase activity was assessed at 48 h post-transfection using the Promega Dual-Luciferase system (Promega, # E1910). The complementary sequences of the 3’UTR reporters of MITF, CREB1 and MAPK1 are as follows:

**Wild-type MITF:** CATGCTTTATCAATAGCCCAGGATATATTTTATTTTTAGAATTTTGTGAAACAGACTTGTATATTCTATTTTACAACTACAAATGCCTCCAAAGTATTGTACAAATAAGTGTGCAGTATCTGTGAACTGAATTCACCACAGACTTTAGCTTTCTGAGCAAGAGGATTTTGCGTCAGAGAAATGTCTGTCCATTTTTATTCAGGGGAAACTTGATTTGAGATTTTTATGCCTGTGACTTCCTTGGAAATCAAATGTAAAGTTTAATTGAAAGAATGTAAAGCAACCAAAAAGAAAAAAAAAAAGAAAGAAAGAGGAAAAGAAATCCATACTAACCCTTTTCCATTTTATAAATGTATTGATTCATTGGTACTGCCTTAAAGATACAGTACCCCTCTAGCTTTGTTTAGTCTTTATACTGCAAACTATTTAAAGAAATATGTATTCTGTAAAAGAAAAAAAAAATGCGGCCTTTTCATGAGGATCGTCTGGTTAGAAAACATAACTGATACCAACCGAAACTGAAGGGAGTTAGACCAAGGCTCTGAAATATAAAGTCTAATCTTGCTCTCTTTTATTCTGTGCTGTTACAGTTTTCTTCATCAATGAGTGTGATCCAGTTTTTCATAAGATATTTTATTTTGAAATGGAAATTAATGTCCTCTCAAAGTAAAATATTGAGGAGCACT

**Mutant MITF:** CATGCTTTATCAATAGCCCAGGATATATTTTATTTTTAGAATTTTGTGAAACAGACTTGTATATTCTATTTTACAACTACAAATGCCTCCAAAGTATTGTACAAATAAGACTCAACGTGAGTACAAGACAATTCACCACAGACTTTAGCTTTCTGAGCAAGAGGATTTTGCGTCAGAGAAATGTCTGTCCATTTTTATTCAGGGGAAACTTGATTTGAGATTTTTATGCCTGTGACTTCCTTGGAAATCAAATGTAAAGTTTAATTGAAAGAATGTAAAGCAACCAAAAAGAAAAAAAAAAAGAAAGAAAGAGGAAAAGAAATCCATACTAACCCTTTTCCATTTTATAAATGTATTGATTCATTGGTACTGCCTTAAAGATACAGTACCCCTCTAGCTTTGTTTAGTCTTTATACTGCAAACTATTTAAAGAAATATGTATTCTGTAAAAGAAAAAAAAAATGCGGCCTTTTCATGAGGATCGTCTGGTTAGAAAACATAACTGATACCAACCGAAACTGAAGGGAGTTAGACCAAGGCTCTGAAATATAAAGTCTAATCTTGCTCTCTTTTATTCTGTGCTGTTACAGTTTTCTTCATCAATGAGTGTGATCCAGTTTTTCATAAGATATTTTATTTTGAAATGGAAATTAATGTCCTCTCAAAGTAAAATATTGAGGAGCACT

**Wild-type CREB1:** AGGAGCATCTCAGAGAAGTGAGAGTAAATCTGAGTTAGCTTAAAAATTGGTAGGGAGGAAGAAAATCTCTGCAAATAATGATTTTATGTTTGTTGGCCAAGTGAAATGATCTATCATTGTGTTTGGGAGGTTTTATTTTCTTATGTTTTTAAAATTGGTAAATGCTTTATAGATGTATTTTTATCCAAGTGCCACTCCAATTTGTGTATGTAATAAAATTATTTATATTAAAAGTGGGAAATAATTGTCAACATTTTTTTTGAGTATAGATTTATTAGGGGTGGCAAAGAAGAGTGCTAGTTAGCAGTTTTCCATGTAAAGTTGTCCTTGACTGATTTGTCCACATGTCAGTTGTAACTCCCCCACTCCCTGCAAAAGGAATTATTTCTAACCCAGATGTATCACTTGAAACTTTTTAGAAGCAAAATAATCAGGGAAGTTCCTAGAAAGGTGTTTGGCTTTTTGGTTTTTGAGGGTTGGGGTAAAGAAGACTTCCCCCA

**Mutant CREB1:** AGGAGCATCTCAGAGAAGTGAGAGTAAATCTGAGTTAGCTTAAAAATTGGTAGGGAGGAAGAAAATCTCTGCAAATAATGATTTTATGTTTGTTGGCCAAGTGAAATGATCTATCATTGTGTTTGGGAGGTTTTATTTTCTTATGTTTTTAAAATTGGTAAATGCTTTATAGATGTATTTTTATCCAAGTGCCACTCCAATTTGTGTATGTAATAAAATTATTTATATTAAAAGTGGGAAATAATTGTCAACATTTTTTTTGAGTATAGATTTATTAGTCTGTGCAAAGAAGACATGACCTTAGCAGTTTTCCATGTAAAGTTGTCCTTGACTGATTTGTCCACATGTCAGTTGTAACTCCCCCACTCCCTGCAAAAGGAATTATTTCTAACCCAGATGTATCACTTGAAACTTTTTAGAAGCAAAATAATCAGGGAAGTTCCTAGAAAGGTGTTTGGCTTTTTGGTTTTTGAGGGTTGGGGTAAAGAAGACTTCCCCCA

**Wild-type MAPK1:** TGCTGACCATGCAGCCGCACCAGAGAGAGATTCTTCCCCAATTGGCTCTAGTCACTGGCATCTCACTTTATGATAGGGAAGGCTACTACCTAGGGCACTTTAAGTCAGTGACAGCCCCTTATTTGCACTTCACCTTTTGACCATAACTGTTTCCCCAGAGCAGGAGCTTGTGGAAATACCTTGGCTGATGTTGCAGCCTGCAGCAAGTGCTTCCGTCTCCGGAATCCTTGGGGAGCACTTGTCCACGTCTTTTCTCATATCATGGTAGTCACTAACATATATAAGGTATGTGCTATTGGCCCAGCTTTTAGAAAATGCAGTCATTTTTCTAAATAAAAAGGAAGTACTGCACCCAGCAGTGTCACTCTGTAGTTACTGTGGTCACTTGTACCATATAGAGGTGTAACACTTGTCAAGAAGCGTTATGTGCAGTACTTAATGTTTGTAAGACTTACAAAAAAAGATTTAAAGTGGCAGCTTCACTCGACATTTGGTGAGAG

**Mutant MAPK1:** TGCTGACCATGCAGCCGCACCAGAGAGAGATTCTTCCCCAATTGGCTCTAGTCACTGGCATCTCACTTTATGATAGGGAAGGCTACTACCTAGGGCACTTTAAGTCAGTGACAGCCCCTTATTTGCACTTCACCTTTTGACCATAACTGTTTCCCCAGAGCAGGAGCTTGTGGAAATACCTTGGCTGATGTTGCAGCCTGCAGCAAGTGCTTCCGTCTCCGGAATCCTTGGGGAGCACTTGTCCACGTCTTTTCTCATATCATGGTAGTCACTAACATATATAAGGTAGGAGATCTTGGCCCAGCTTTTAGAAAATGCAGTCATTTTTCTAAATAAAAAGGAAGTACTGCACCCAGCAGTGTCACTCTGTAGTTACTGTGGTCACTTGTACCATATAGAGGTGTAACACTTGTCAAGAAGCGTTATGTGCAGTACTTAATGTTTGTAAGACTTACAAAAAAAGATTTAAAGTGGCAGCTTCACTCGACATTTGGTGAGAG

To explore transcriptional modulation of CREB1 at the MITF promotor, plasmids encoding the following reporter genes were generated employing pGL3-Basic plasmids: h-MITF-pro, containing 2000 bp of sequence upstream of the transcription start site, which included a CREB1-binding site (-1357 to -1351); and h-MITF-mut, including the mutated binding site (TCTGATG mutated to GCAGCTC). pGL3-Basic was used as a negative control, and the above plasmids together with CREB1 plasmids or the control vectors were respectively co-exposed to transfection into HEK293T cultures. Reporter activity was determined at 48 h post-transfection employing the Promega Dual-Luciferase system.

**Cell Counting Kit-8 (CCK-8) assay**

Cell proliferation was assessed by CCK-8 assays (Beyotime, #C0038). Different cell groups, including duplicate groups, were seeded into 96-well plates at a concentration of 1000 cells/200 μl per well. Absorbance was measured at 450 nm.

**Transwell invasion and migration assay**

After GC cells were transfected or infected with the indicated molecules, 200 μl of cells at a confluence of 1×10^5^/ml were starved in serum-free medium for an additional 12 h and then trypsinized to be seeded in the upper chambers of Transwell 24-well plates (Corning, #3422). In addition, 500 μl of foetal bovine serum (FBS)-containing medium (20% confluence) was added to the lower Transwell chamber. After culturing for 24 h, the cells were fixed in 95% pre-cooled ethanol for 10 min. Non-invasive or non-motile cells on the upper side of the filter were removed; invasive or migrated cells were stained with 0.5% crystal violet for 30 min. Five fields for each well were photographed under a microscope (20×magnification). The invasion assay consisted of an additional step for coating Matrigel (BD, #356243) onto the Transwell chamber membrane before cell inoculation and cells were plated at a confluence of 2.5×10^5^/ml.

**Table 1**

| **Gene Name** | | **Sequence** |
| --- | --- | --- |
| miR-585-5p | | CUAGCACACAGAUACGCCCAGA |
| MITF | -F | AGGCATGAACACACATTCACGAG |
|  | -R | CAGGATCCATCAAGCCCAAGA |
| CREB1 | -F | AGAAACATGGAAAAGGGCAAAC |
|  | -R | AAAAGGGAGAGGGAGGAGGAG |
| MAPK1 | -F | CGTTGGTACAGGGCTCCAGAA |
|  | -R | CTGCCAGAATGCAGCCTACAGA |
