# Supplementary material for: MiR-585-5p impedes gastric cancer proliferation and metastasis by orchestrating the interactions among CREB1, MAPK1 and MITF
Source: Front Immunol. 2022 Oct 4;13:1008195. doi: 10.3389/fimmu.2022.1008195 (PMC9576935; doi:10.3389/fimmu.2022.1008195)
Supplement: Supplementary file 7 [file Table_2.docx]

| **miRNA Name** | [hsa-miR-585-5p](http://mirdb.org/cgi-bin/mature_mir.cgi?name=hsa-miR-585-5p) | | **miRNA Sequence** | CUAGCACACAGAUACGCCCAGA |  |
| --- | --- | --- | --- | --- | --- |
| **miRBase ID** | | [MIMAT0026618](http://www.mirbase.org/cgi-bin/mature.pl?mature_acc=MIMAT0026618) | | | |

| **NCBI Gene ID** | [1385](http://www.ncbi.nlm.nih.gov/entrez/query.fcgi?db=gene&cmd=Retrieve&dopt=full_report&list_uids=1385) | **GenBank Accession** | [NM_134442](http://www.ncbi.nlm.nih.gov/entrez/query.fcgi?cmd=Search&db=Nucleotide&term=NM_134442) |
| --- | --- | --- | --- |
| **Gene Symbol** | CREB1 | **3' UTR Length** | 8944 |
| **Gene Description** | cAMP responsive element binding protein 1 | | |
| **3' UTR Sequence** | | | |


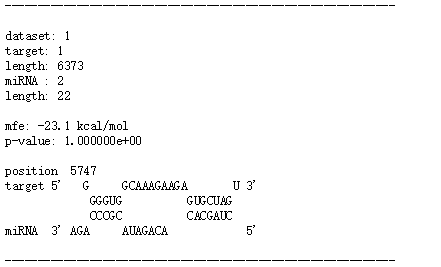


**1、The blue fonds were predicted as the promising binding sites of miR-585-5p based on merging the seed sequence alignments and free energy calculation. The yellow-highlighted sequences were the top-rated which we chose for follow-up detection.**

>hg38_knownGene_ENST00000432329.6 range=chr2:207597059-207603431 5'pad=0 3'pad=0 strand=+ repeatMasking=none

TTTGGGATTTAAATTTTCACCTGTTAAGGTGGAAAATGGACTGGCTTGGCCACAACCTGAAAGACAAAATAAACATTTTATTTTCTAAACATTTCTTTTTTTCTATGCGCAAAACTGCCTGAAAGCAACTACAGAATTTCATTCATTTGTGCTTTTGCATTAAACTGTGAATGTTCCAACACCTGCCTCCACTTCTCCCCTCAAGAAATTTTCAACGCCAGGAATCATGAAGAGACTTCTGCTTTTCAACCCCCACCCTCCTCAAGAAGTAATAATTTGTTTACTTGTAAATTGATGGGAGAAATGAGGAAAAGAAAATCTTTTTAAAAATGATTTCAAGGTTTGTGCTGAGCTCCTTGATTGCCTTAGGGACAGAATTACCCCAGCCTCTTGAGCTGAAGTAATGTGTGGGCCGCATGCATAAAGTAAGTAAGGTGCAATGAAGAAGTGTTGATTGCCAAATTGACATGTTGTCACATTCTCATTGTGAATTATGTAAAGTTGTTAAGAGACATACCCTCTAAAAAAGAACTTTAGCATGGTATTGAAGGAATTAGAAATGAATTTGGAGTGCTTTTTATGTATGTTGTCTTCTTCAATACTGAAAATTTGTCCTTGGTTCTTAAAAGCATTCTGTACTAATACAGCTCTTCCATAGGGCAGTTGTTGCTTCTTAATTCAGTTCTGTATGTGTTCAACATTTTTGAATACATTAAAAGAAGTAACCAACTGAACGACAAAGCATGGTATTTGAATTTTAAATTAAAGCAAAGTAAATAAAAGTACAAAGCATATTTTAGTTAGTACTAAATTCTTAGTAAAATGCTGATCAGTAAACCAATCCCTTGAGTTATATAACAAGATTTTTAAATAAATGTTATTGTCCTCACCTTCAAAAATATTTATATTGTCACTCATTTACGTAAAAAGATATTTCTAATTTACTGTTGCCCATTGCACTTACATACCACCACCAAGAAAGCCTTCAAGATGTCAAATAAAGCAAAGTGATATATATTTGTTTATGAAATGTTACATGTAGAAAAATACTGATTTTAAATATTTTCCATATTAACAATTTAACAGAGAATCTCTAGTGAATTTTTTAAATGAAAGAAGTTGTAAGGATATAAAAAGTACAGTGTTAGATGTGCACAAGGAAAGTTATTTTCAGACATATTTGAATGACTGCTGTACTGCAATATTTGGATTGTCATTCTTACAAAACATTTTTTTGTTCTCTTGTAAAAAGAGTAGTTATTAGTTCTGCTTTAGCTTTCCAATATGCTGTATAGCCTTTGTCATTTTATAATTTTAATTCCTGATTAAAACAGTCTGTATTTGTGTATATCATACATTGTTTTCAATACCACTTTTAATTGTTACTCATTTTATTCACTAAGCTCGATAAATCTAACAGTTACTCTTAAAAAAAAAAAAAAAAGACTAAGGTGGATTTTAAAAATTGGAAACTGACATAATGTTAGGTTATAATTTCTCATTTGGAGCCGGGCGCAGTGGCTCACGCCTGTAATCCCAGCACTTTGGGAGGCCAAGGTGGGTGGATCACCTGTGGTCAAGAGTTCAAGACCAGCCTGGCCATCATGGTGAAACCCCATCTCTACTAAAAATACAAAAATTAGCCAGGCGTGGTGGCTGGCGCCTGTAATCCCAGCTACTCAGGAGGTTGAGGCAGCAGAATTGCTTGAACCCAGGAGGCAGAGGGTTGCAGTGAGCCGAGATAGCACCATTGCACTCCAGCCTGGGCGACTCCATCTCAAAAAATAAAAATAAAAAAAATGTCTCATTTGGGAAGGAAATTCCTTTTAAAAAAGAGTTGAGACACTTAGAAAACTAATGTTTTATATTTAGTCAAGAGTTATTTAAGAAAGTCAAGCTTGTTTAACAACAAAATATGAAGATTTAAGTGTTAATTGCTGGATCCATTTTAAAATAAGATTTTAATTAACATTTGTAAATGGTATATTTTCGTTTGTAACAAACCATTGTCTTTTTTCAAGGATGAACAGAGTTTATGAAGGAGCATCATTCTAAGAATTAAGTGATGTAGTCTTTATGTTTGGACAGTTCACCAGATTCTCAAGAAGGCTTTCAAACAACTATAAAGTTTGATGTTTGTCCTGCTGAGCTAATGGGGAAAGTTATAGCATAAAAATTGTGTAACCGCATAGATATGTCATTTTTAAAAACTGGTTTAACAGAAATCAAGCAAAGTCACAAATATGTTCACAAGTTGGAATTATTTATTGAGTCAAAATGTCGAATCGAACATTTTGAATGAAGTAAGTGTTATAAATGAAAAATTGCCTGATGTTTAGCAGTTTGTATTCTCTAAAGCTTTTTTTCAAAAGTTCAGGCTTTCTACTTACTGGGAAGTTGGTGGTCCTCTTAGTCCCTGATAAATCAAGGCAATCACATTCATGTGAGCTGGATGAATTTATAAGTTATAAAGACCTTATCCTTCATACCTTGAGGATGATTGCACTGGTTTTGAAGTCAGTTGCTTAATGATGAGGTGAGAAATGTATCCTGTTGCTAAATCTGTCTTAGACCCTTGGTGAAACTTGAAGATTTCAGTTTATAAAGATAAAATCAAGCATCTTTTGTGCAGTTTTCTTTTTTTAATGCAAGAATGGTGGGGAGGTTTGTTTGTAAGCATGAAACTTTGAGAATCTTTATTAAGAAAATGACATAATTTTTAAAAACCTTGTAGCCAAGAACATATGTGGCCACATTACCAGTAATAAATGTTTTTCTCTTTATATTGGCCAAAAGGGAATAAAAATGTCATCATAGGAATTTGTACATATGCTACTGATTTGCCTAGAAAATAGCAAGTTTGATATTGCTCACTTTGCAAATATAGGGCCATGTGGCACTTTTATCTATAGGACAGATTAATAAAAATGAAGTGGGGAGGGGTTTATTTTTGATATATTACTCTTATGAGTTTTCAAGCTTTGATAATGTTTAACTGAAAAGTGGCTTAGAAAGGGCTAGATCCAATGTGTTCATTATTAAATAATTGCTATCAGATACAATTTTAAGTTCATTCTTTTTCAAACTCAAGTACCATATTGGCAACCATAATATTGTCATAGGTGCTCTCTTCATTTAGATATTCTTGGGGGGGGTGGCATTTGTATAATATATGTGTACATATATATATATATATATATATATACATACAGTATATAATCTAAAGCTCTGAGAGCTCTTAAGTCAGGAATGCTGAGTATTATAGTATATTGAGGTCAGATGAAATTTTACATTTTTGTGTGTTCTGTTGCATTCCTTCTGGTAGTTTCTATGACTGCATTACTCCAGCACTCATGATTGATTTTATCTTCTAATTTTCTTCCAAGTATTTTATTTTTTATTAGTTTTCTTTGGCTTGATACTTTTAAATATGTTACTAGTCACTTGAAAGCCTCTCCCCCAAAAGTATTTGGTTTGTATGCTTTGTCTGTGGCAGCTATAACAGTGGTAAGAACATTTTGAAGATAGCTTTTTAAAGGAACCACTGATTTTTTCAAAAATCATCCTGGGGGAGGAATTTTGGCATTTCATTTGAGCAGGGATTTTGTCAGAAAATGTGTTTTGATGGTAGGTCAGCAGCAGTGCTAGTCTCTGAAAGCACAATACCAGTCAGGCAGCCTATCCCATCAGATGTCATCTGGCTGAAGTTTATCTCTGTCTCTCAGGATAAATCCCTGTAGGACAAATCCCTACTATCATTTCTACCTTTTGGGGTGACATGTGGAATCATACAAAGGCTTAGGAAGAAATACGTTTGTTTAAACCAGGATGCTTTACTTACTTGAAGTGACTTCAATCTAGATTTCTTTTAATATTTAACAAATTTTTAATTCTATGATCAGCCACAGTCAGCTATTACCATAAATTGGTCTCTGTTTATTTTGAAGATCACGGCTGCTTCATTTTGCAGGATTAAGTAGGGCTAATGTATCTTAAAGTTAAGATCTTGAATTAAAGTGAGTTTTAGAAATAGTGTTACATACCTTTTCAGTTGTTTTCAAGAGGCTTTATTTTTGTTGCCTTTGTAGCCCTGAAAGCTGTTGGTATATTTTTTCCCTCATGGACCCAATAGAAAAGTTGTATATTTATTTGGATTATATTTACATTCTGTCCTTTGTAAATGTTTGGTGTAACTTGCACTTTTTTAAATGACCCAGTTTGGGTATTAGCAACTTAAGAAATTCCCTCATCAAGTAATTCTCAACTTTTTAGTCTTTCTCCTCTCTTCAAATCATGTGACTTTTTAAATGGAAGTTTTTCATTGATTAAAATATTTTAGCACCTAAAAGCTAGCCTTAAAAACAGCTGTAAAAGAAAAACATCAGGAAATTAGATATGACTAGCCCAGTTAATTAAAAGACGGGCTCAAACCTTGTTTTATTCTTTTTCATCTTGGATGAAGATTGAAGGGAAAATAACTCAAGTGCATAATATTTATTTTCAATTTTTAATGAGACTTTATCCTCATCACAACATTAATACTGTACATAGTATGCCAAAATATCCATTAATTTGTCTAGAATAGTACAAGACTTTTTAAAGCAATTGTCCTCACAGAGACCACATGTAATATACTGAAATATGTTCATTTTTAATGGCTTTGTTAACATCAAAGAAATGCTGCCTAAATTTGATTTCAGATGAGGAAGGAGAAAGTAAAGTGTGCATAGTAAGGCTGTAGGTGAAGAGTTGTGAGATAAATAGTTCACTCAGTTGTACAAAGCACAACTAGAACTTTTTGTTGGGAGGCTTACATACATCTTGAATATTCTTAATGTAATAATGTTGACTATTAAGTTGGCTACACAGTCACTGTATGTACTAGGAACTGGTTTCCTTGACATTCTAGAATCAATGGCTAGGAGAGGCATTAATCTTTGAGGGGCTGAACATATCATGAAGCTGAGTCAGTATGGAAAATTTTCAAATAAACAGGGTGCTGAAGTTCCATCTGTCTCATCTGCTTATGATAAGTTCTTATTGATTAGTGAATGTAGCTTAAGCCTTTGTATGTGTCCTCAGGGGGCAGACCGACTTTAAGAGGGACCAGATAACGTTTGAATGGAGGGATTATATTTCAGGTGTTTTAGCTTGAAATTTATTTTTTAAAAAAAGAAAAATTTAAAAAATATATAAATAAAATAGAACAAAGCCGGTGATGCAAGTTGATATTATAAACAGGCAGTTTTAGCACAGAAAGAAAATACTGACCTGTCTGCATTCTGGTACGGTGGGTGCAGGTCCCAGCTGGGTATGACATGATACATTTTTAATTATTCTCACCAGCAAGTAAAAGGAAAATGAACAATCTTTTGGAATTGTCTTTGAAAAGGATCAAAGAGTAGGAAATTCACATTTGACCTAACATTACTTGCCTATAGAAGTATGGCATTTCCAAGCTTTTGTCTGAGGAGCATCTCAGAGAAGTGAGAGTAAATCTGAGTTAGCTTAAAAATTGGTAGGGAGGAAGAAAATCTCTGCAAATAATGATTTTATGTTTGTTGGCCAAGTGAAATGATCTATCATTGTGTTTGGGAGGTTTTATTTTCTTATGTTTTTAAAATTGGTAAATGCTTTATAGATGTATTTTTATCCAAGTGCCACTCCAATTTGTGTATGTAATAAAATTATTTATATTAAAAGTGGGAAATAATTGTCAACATTTTTTTTGAGTATAGATTTATTAGGGGTGGCAAAGAAGAGTGCTAGTTAGCAGTTTTCCATGTAAAGTTGTCCTTGACTGATTTGTCCACATGTCAGTTGTAACTCCCCCACTCCCTGCAAAAGGAATTATTTCTAACCCAGATGTATCACTTGAAACTTTTTAGAAGCAAAATAATCAGGGAAGTTCCTAGAAAGGTGTTTGGCTTTTTGGTTTTTGAGGGTTGGGGTAAAGAAGACTTCCCCCACAACTGTCAGCACAAAACAGGGTATTGATTTTTAACTCTGATGTTTCTATTGGAGTTGAATACTAAATAAATAACTATAATGAGGGAAATACATTTCTAATAAAATTCCCTACATTCTAGAAACATCCCTGTTTTAATTTTTTTATCTAAATCTTTTTGTGCTTTATGTGTAAAGAAAAAAATGTACTGAGTTACAATGCATTTTATTAACACTATGTACATAATAGCTGCTTTGTGTTCAGAATAGTAGCAGTTGCTTTGTATATTAAAGTGATCCTTGTGAATTTGTGAAATATTGTCATAAAGTGCTTTTTCTTACTGTAATCTTTGTGGTATCAACTGTCATAATGCTCTTTTTACACAAACATTTATGTGCAGTCACATAAACATGCTTTTAAAAACTC

**2、The blue fonds were predicted as the promising binding sites of miR-585-5p based on seed sequence alignments. The yellow-highlighted sequences were the top-rated which we chose for follow-up detection.**

| **NCBI Gene ID** | [5594](http://www.ncbi.nlm.nih.gov/entrez/query.fcgi?db=gene&cmd=Retrieve&dopt=full_report&list_uids=5594) | **GenBank Accession** | [NM_002745](http://www.ncbi.nlm.nih.gov/entrez/query.fcgi?cmd=Search&db=Nucleotide&term=NM_002745) |
| --- | --- | --- | --- |
| **Gene Symbol** | MAPK1 | **3' UTR Length** | 4593 |
| **Gene Description** | mitogen-activated protein kinase 1 | | |

>hg38_knownGene_ENST00000215832.10 range=chr22:21754500-21769203 5'pad=0 3'pad=0 strand=- repeatMasking=none

ATTTGTCAGGACAAGGGCTCAGAGGACTGGACGTGCTCAGACATCGGTGTTCTTCTTCCCAGTTCTTGACCCCTGGTCCTGTCTCCAGCCCGTCTTGGCTTATCCACTTTGACTCCTTTGAGCCGTTTGGAGGGGCGGTTTCTGGTAGTTGTGGCTTTTATGCTTTCAAAGAATTTCTTCAGTCCAGAGAATTCCTCCTGGCAGCCCTGTGTGTGTCACCCATTGGTGACCTGCGGCAGTATGTACTTCAGTGCACCTACTGCTTACTGTTGCTTTAGTCACTAATTGCTTTCTGGTTTGAAAGATGCAGTGGTTCCTCCCTCTCCTGAATCCTTTTCTACATGATGCCCTGCTGACCATGCAGCCGCACCAGAGAGAGATTCTTCCCCAATTGGCTCTAGTCACTGGCATCTCACTTTATGATAGGGAAGGCTACTACCTAGGGCACTTTAAGTCAGTGACAGCCCCTTATTTGCACTTCACCTTTTGACCATAACTGTTTCCCCAGAGCAGGAGCTTGTGGAAATACCTTGGCTGATGTTGCAGCCTGCAGCAAGTGCTTCCGTCTCCGGAATCCTTGGGGAGCACTTGTCCACGTCTTTTCTCATATCATGGTAGTCACTAACATATATAAGGTATGTGCTATTGGCCCAGCTTTTAGAAAATGCAGTCATTTTTCTAAATAAAAAGGAAGTACTGCACCCAGCAGTGTCACTCTGTAGTTACTGTGGTCACTTGTACCATATAGAGGTGTAACACTTGTCAAGAAGCGTTATGTGCAGTACTTAATGTTTGTAAGACTTACAAAAAAAGATTTAAAGTGGCAGCTTCACTCGACATTTGGTGAGAGAAGTACAAAGGTTGCAGTGCTGAGCTGTGGGCGGTTTCTGGGGATGTCCCAGGGTGGAACTCCACATGCTGGTGCATATACGCCCTTGAGCTACTTCAAATGTGGGTGTTTCAGTAACCACGTTCCATGCCTGAGGATTTAGCAGAGAGGAACACTGCGTCTTTAAATGAGAAAGTATACAATTCTTTTTCCTTCTACAGCATGTCAGCATCTCAAGTTCATTTTTCAACCTACAGTATAACAATTTGTAATAAAGCCTCCAGGAGCTCATGACGTGAAGCACTGTTCTGTCCTCAAGTACTCAAATATTTCTGATACTGCTGAGTCAGACTGTCAGAAAAAGCTAGCACTAACTCGTGTTTGGAGCTCTATCCATATTTTACTGATCTCTTTAAGTATTTGTTCCTGCCACTGTGTACTGTGGAGTTGACTCGGTGTTCTGTCCCAGTGCGGTGCCTCCTCTTGACTTCCCCACTGCTCTCTGTGGTGAGAAATTTGCCTTGTTCAATAATTACTGTACCCTCGCATGACTGTTACAGCTTTCTGTGCAGAGATGACTGTCCAAGTGCCACATGCCTACGATTGAAATGAAAACTCTATTGTTACCTCTGAGTTGTGTTCCACGGAAAATGCTATCCAGCAGATCATTTAGGAAAAATAATTCTATTTTTAGCTTTTCATTTCTCAGCTGTCCTTTTTTCTTGTTTGATTTTTGACAGCAATGGAGAATGGGTTATATAAAGACTGCCTGCTAATATGAACAGAAATGCATTTGTAATTCATGAAAATAAATGTACATCTTCTATCTTCACATTCATGTTAAGATTCAGTGTTGCTTTCCTCTGGATCAGCGTGTCTGAATGGACAGTCAGGTTCAGGTTGTGCTGAACACAGAAATGCTCACAGGCCTCACTTTGCCGCCCAGGCACTGGCCCAGCACTTGGATTTACATAAGATGAGTTAGAAAGGTACTTCTGTAGGGTCCTTTTTACCTCTGCTCGGCAGAGAATCGATGCTGTCATGTTCCTTTATTCACAATCTTAGGTCTCAAATATTCTGTCAAACCCTAACAAAGAAGCCCCGACATCTCAGGTTGGATTCCCTGGTTCTCTCTAAAGAGGGCCTGCCCTTGTGCCCCAGAGGTGCTGCTGGGCACAGCCAAGAGTTGGGAAGGGCCGCCCCACAGTACGCAGTCCTCACCACCCAGCCCAGGGTGCTCACGCTCACCACTCCTGTGGCTGAGGAAGGATAGCTGGCTCATCCTCGGAAAACAGACCCACATCTCTATTCTTGCCCTGAAATACGCGCTTTTCACTTGCGTGCTCAGAGCTGCCGTCTGAAGGTCCACACAGCATTGACGGGACACAGAAATGTGACTGTTACCGGATAACACTGATTAGTCAGTTTTCATTTATAAAAAAGCATTGACAGTTTTATTACTCTTGTTTCTTTTTAAATGGAAAGTTACTATTATAAGGTTAATTTGGAGTCCTCTTCTAAATAGAAAACCATATCCTTGGCTACTAACATCTGGAGACTGTGAGCTCCTTCCCATTCCCCTTCCTGGTACTGTGGAGTCAGATTGGCATGAAACCACTAACTTCATTCTAGAATCATTGTAGCCATAAGTTGTGTGCTTTTTATTAATCATGCCAAACATAATGTAACTGGGCAGAGAATGGTCCTAACCAAGGTACCTATGAAAAGCGCTAGCTATCATGTGTAGTAGATGCATCATTTTGGCTCTTCTTACATTTGTAAAAATGTACAGATTAGGTCATCTTAATTCATATTAGTGACACGGAACAGCACCTCCACTATTTGTATGTTCAAATAAGCTTTCAGACTAATAGCTTTTTTGGTGTCTAAAATGTAAGCAAAAAATTCCTGCTGAAACATTCCAGTCCTTTCATTTAGTATAAAAGAAATACTGAACAAGCCAGTGGGATGGAATTGAAAGAACTAATCATGAGGACTCTGTCCTGACACAGGTCCTCAAAGCTAGCAGAGATACGCAGACATTGTGGCATCTGGGTAGAAGAATACTGTATTGTGTGTGCAGTGCACAGTGTGTGGTGTGTGCACACTCATTCCTTCTGCTCTTGGGCACAGGCAGTGGGTGTAGAGGTAACCAGTAGCTTTGAGAAGCTACATGTAGCTCACCAGTGGTTTTCTCTAAGGAATCACAAAAGTAAACTACCCAACCACATGCCACGTAATATTTCAGCCATTCAGAGGAAACTGTTTTCTCTTTATTTGCTTATATGTTAATATGGTTTTTAAATTGGTAACTTTTATATAGTATGGTAACAGTATGTTAATACACACATACATACGCACACATGCTTTGGGTCCTTCCATAATACTTTTATATTTGTAAATCAATGTTTTGGAGCAATCCCAAGTTTAAGGGAAATATTTTTGTAAATGTAATGGTTTTGAAAATCTGAGCAATCCTTTTGCTTATACATTTTTAAAGCATTTGTGCTTTAAAATTGTTATGCTGGTGTTTGAAACATGATACTCCTGTGGTGCAGATGAGAAGCTATAACAGTGAATATGTGGTTTCTCTTACGTCATCCACCTTGACATGATGGGTCAGAAACAAATGGAAATCCAGAGCAAGTCCTCCAGGGTTGCACCAGGTTTACCTAAAGCTTGTTGCCTTTTCTTGTGCTGTTTATGCGTGTAGAGCACTCAAGAAAGTTCTGAAACTGCTTTGTATCTGCTTTGTACTGTTGGTGCCTTCTTGGTATTGTACCCCAAAATTCTGCATAGATTATTTAGTATAATGGTAAGTTAAAAAATGTTAAAGGAAGATTTTATTAAGAATCTGAATGTTTATTCATTATATTGTTACAATTTAACATTAACATTTATTTGTGGTATTTGTGATTTGGTTAATCTGTATAAAAATTGTAAGTAGAAAGGTTTATATTTCATCTTAATTCTTTTGATGTTGTAAACGTACTTTTTAAAAGATGGATTATTTGAATGTTTATGGCACCTGACTTGTAAAAAAAAAAAACTACAAAAAAATCCTTAGAATCATTAAATTGTGTCCCTGTATTACCAAAATAACACAGCACCGTGCATGTATAGTTTAATTGCAGTTTCATCTGTGAAAACGTGAAATTGTCTAGTCCTTCGTTATGTTCCCCAGATGTCTTCCAGATTTGCTCTGCATGTGGTAACTTGTGTTAGGGCTGTGAGCTGTTCCTCGAGTTGAATGGGGATGTCAGTGCTCCTAGGGTTCTCCAGGTGGTTCTTCAGACCTTCACCTGTGGGGGGGGGGGTAGGCGGTGCCCACGCCCATCTCCTCATCCTCCTGAACTTCTGCAACCCCACTGCTGGGCAGACATCCTGGGCAACCCCTTTTTTCAGAGCAAGAAGTCATAAAGATAGGATTTCTTGGACATTTGGTTCTTATCAATATTGGGCATTATGTAATGACTTATTTACAAAACAAAGATACTGGAAAATGTTTTGGATGTGGTGTTATGGAAAGAGCACAGGCCTTGGACCCATCCAGCTGGGTTCAGAACTACCCCCTGCTTATAACTGCGGCTGGCTGTGGGCCAGTCATTCTGCGTCTCTGCTTTCTTCCTCTGCTTCAGACTGTCAGCTGTAAAGTGGAAGCAATATTACTTGCCTTGTATATGGTAAAGATTATAAAAATACATTTCAACTGTTCAGCATAGTACTTCAAAGCAAGTACTCAGTAAATAGCAAGTCTTTTTAAATGCTGCTTTATTTCACTAAATTTTGTTGTGAGGTGTCACTAAAATGCCTGCAAACAAACGTAACTGCTAATCTGAGAGGAAACCCTCTTACTAATCAGAGAAGAAACCCTCCTGTCAGAAACCTTCAGGGAAGTGAGCTGATCACACCTAAACTGGGAGTTTGCAATGGGGTATTTGAAGCACTGTGGGAGTATTCCACTGGCCCTCTCCCTGAGAGACTTAACAGTCTTCCCTGTTGTCCAGATTCTGTATAAGGCAATCAGAATAATCATCTTCCTTGTTCAGCAGAGGAGCCTGGTCCCATTTTCCCCACTTTGTGATGGGCTTCTCTCAGCGGTAGCTCAGCAGTTCCAGATGGCAGTTTGGACCAGCATCTAGGCTGGCCAGTTCGCTGTGTTTACTTAGAACCAACACGTTCAGAGCTGGCCTGGACCATCTGAGGGGAACAGGAAACACCCCTAGGCTGTGGAAGCAAGTGCAGACCCCCACCCCCGGCCCTGAAGCCAAGGGGGCAGGGTTTGGGAGTGGCCAAAGAGAAGCAGTGCAGGGATGGGTTTTCCTAGGGACAGGCTTAGCATTCCTGACTCTAGGAAGAAGGAGCAGTGAGGCGGAGAAACAGTGGAGGGGATGGTGGCATTGGGCCCCATGGGGCCGAGATGGACACAGGGCTCGTTCTCTTGAGTCTGGTGCCAAGGACAGCTGAAGACGACATCATTTTCAGGTGGAGAGGAGAGAGTGGAGGGAGATCATGCCCTGTGATGTGTCTTTTGCAGGTGAAGGTGGGAGACAAGGTCTCTGCTGACGATGAGGCAGAGCCACCGTGAAAGTTGTAATAGGAGGACTGCCCGCCGCTGGAAGGGCCTGCAGTGACGCTAGGACACCCTCTGCCTGCATGTCACGTTAGCTGGGCTGGGCGAAGTAGAAGACCAAGGGGAAGAGGTGCAGTGGGGAGACCAGGTGGGATGCAACCACAGGACCAGTGGAGGGGCTGTGGCACGTGGGCGGAGACTGAGTGGCTGGGCATGTGTTGTGGCTGAGCATGTGGTGTGGGCAGTGGTCCTAGACCCCGCCATGTCCGGACAATGATATAGAGCGTCTCAGCATCGCCAGTCTAGACTGTCTATGGAGAGCAGAAAGTTGTCTGGGGCTGCCTGGGGAACTGTGAGGCCAGCTATATCACCGTCGCTGATGGTGACATTACGGTGGTGGCAGGAGCAAGGAGAGAGGGAAGAAGGACCCCGTCCAGCTTTAGTCACAAAATACCCAATGGAAGATGCCAGTGCCAATCCTGTGGGTTTCCTTGGGACTTCACACTGGCTTTCTTATCTGCTCCAGATCCATTCAGTAGTCACTGAGTTCCTGCCAAATACTTTGTAGCGCCAGAAGCCAGGAGCGGGGTCTGCAGCAGGGCAGTCCCCATTTTCAGGAAATGCCTGGAGCTGCTGGTCCCTGAGAGAAAGGAAAACATCTTTCAGCCGTACGCAGGCCAAGAAGGCCAATGTCCAGTAGCTTTGTGATTTTTTTTATATTTTTTTATTTATTTATTTTTGAGATAGAGTCTTGCTCTGTCGCTCAGGCTGGAGTGCAGTGGCGTGATCTCCACTCACTGCAACTTCCGCCTCCTGGGGTCAAGCAGTTCTGCCTCAGCCTCCCGAGTAGCTGGGATTACAGGCACACGCCACCACACCCAGCTAATTTTTGTGTTTTTAGTAGAGACGGTTTCACCATGTCGGCCAGGCTGGTCTCAAACTCCTGACCTCAGATGATTCAGCCTCCCAAAGTGCTGGGATTACAGGTGTGAGCCACTGCACCCAGCCTGTGATGTTTCTGTGGGGTTCCACAAATGTGTGTGTGTGTAAAAGCTGATGATTACAGCAAGAATGTGAACAGTAGCAGTTTTCCATTTGAAGGCAAGTTTTGTCTTTATCTGGGTATCAGAAGGACCCTCTGGGCCATTGTCGCTTCCTGTACTCAGAGCCACCCTAGTACTACGGGCACACACAGAAAACAGCAGCCTGCGTACTTTCAAAGGAAAGGCATCTTTAATCACCAATGCCTGGAAAAATTATTTTGTTTCCCTCTTCCTTCCGTCTTGTTTCCTAACTTCTTACCAAAGTTTAGAGTCTGAGTTTTTCGTATAATAATGTCCCACATCCACACATCGGGCCTACAGATGCTCTCCCTTGAATCGACTGGAAACATGACACCGGTTCCATGCTCTGGAACTGTCACCTGTGATGTGCTGGGCTGTGTCCCAAGCACAGGAATCCCAGCAGTTTCAGCTCGATGCAGAACCACCATGCTCCAGACACAGGCTTGGGAAAGACACGTCAAAATTAAAATACTAGGTAAGAGAAGCACCTGATTGGGTAGAAGTTGGAGAGGAATCCTGGAATTTTGTGGCCAGAAGGAGCCACTGCCCCTTTTGTTTAGTAAGACTAGACAGTAACAGAAGCCAGTTGTCAGCTATGAAAGTGGTGGGTGAAGCAGGGGAGGCTCCTCTATGGTGGGACCCTGGACAAGGGAAGCCGAATGTGTGAAGAAGGGGTGCGGGGGTGTGCGGTGCCCTAGGACACTAGGGCAAAGGTTTCAAACCTGGAACAAGGCACTGGAGGAAGATCTGCTGCCAGTCAGCAGTGCGGGCCCTCGAGTTAGCAGTCCGTGCGCAGAGGGGCCAGTTCTGAGACCAGTTTGGAGAGTCAGGCAGTGACCCATTGGCCATGTCATAATTCCTTCAGCCTGCCTCCTCTTTAATCCCAGAGAGTGCTCTTTCTTCATACTTCCTTTAAAATACTAAATTGTTCCCATTCCATGGGGAGCTGGCTAGGCTTTACAGGCTAGGAAATGTAGGTTTTCTGAGATGGAACCATCTACACAAGGAGGAGGAAGGCACTAAGACTACAGATGAGACCCATGACAGGGCTGAGCATTTGGAAGCCAACCCTGGTTGCTTTTCAAGAATTGCTTTGTGGCTGGGTGCAGTGGTTCACACCTGTAATTGCAGCACTTTGGGAGGCTGAGGCAGGTGGATTGCTTGAACCCAGGAGTTCGAGACCAGCCTGGGCAACATATGGGACACCCCACCGCCCCCGGCTCTGCAAAAAAATTAAAAATTAGCCAGGCGTGGTGTCATGAGCCTGTGGTCTCAACTACTCAGGAGGCTGAGGCTGGAGGATCGCTTGAACCTGGGAGGTCAAGGCTCCAGTGAACCATAATTGTGCCACTGCACTGCAGCCTGGGCGACAATTTGTTTTCTAAATTGCTTTTGAAAGTCTACTGCATTACATATTCCAAAAAGCAGTGGTTTTCAAATACTTTTATCACCGATATCCTTTTATGAAATGAAATCAGTAGAACTTTCTCTGCTCTGAATAAGCAAGGGTGGGAACCTGTCTACCTCCCACAGATAGCATAATGTGCCTGCCATAGAGGAGCCAAAAAATGGTGATGGGAACTGAGAGGAGAGCAAATGTCACAAAAGACTGAGCAATTGAGAAAACAAAACAAGACCACAGATGACTGTTAACGCCTCCACAGTGGACCAAGAAAGGACAGAGAGCTGGCAGCATGGGCATCACTGTCTGGTCGGCAGCAGGAAGGCCTCGCTAGGGAATTGAGTACAGTCATCTAACTAGTTTAAAAGTACAGGAAGGATGATTAAGGCTATTGGAGAGGTCATACAAATAGGGGAGGGGCAGGCAATGGCTGATAAGACATGAATTTGTAAGGCGATGAGTATTGCAGTCAGCAAAACAAACGAGACTGCTCTCCCAACACATAACTCAGCAGGGAGGCCAGGCATTGGTTTAACCATTTAATATAAAGAAGTTAAAATTACAAATGCGCTAAGTGCCTAAAGAAGAATAAGTGCAGGAATGAGAGCAGCATGGACTGCCACAGTTTTAGAATAAGCACTGTCACTGCTAGATTGGAAACAAAAATCCATAAATTTGGCCCGGTGTGGTGGCGGACGCCTGTAGTCCCAGCTACTTGGAGGCTGAGGCGTGAGAATCGCTTGAACCCGGGAGGCGGAGGTTGCAGTGAGCCGAGATGGCGCCACTGCACTCCAGCCTGGGCGTCAGAGTGAGAACTCTGTATCAAAAAATAAAAATAAAAAAAAGTCCATAAATCTGCAATGTCTCAGTTAAGAAAGAAAGACTGGGCCAATGCAGATTTCAAACCGGAGAAAGTCATACTGTCAGTGAAGGCCGCCTGTGGCCGGAAGGCGCCAGGGGATTAGCACCCTGGACTCAGTGTTGCTGGGAAACAGGGCCCCAAGGCTGGGAGCACAGTGTTTAAAGGGCATCTACCCAAGAAGGGAGCACAGGGCAAGGAGGAGCTGCAGGGGGGCTTGGCTGCCAAAGTGAATTCTGAGGAGAGAGCTATTGCTGCCTACGATATGCAGGCTGCACAGAACACAAGTGGAATCAGCAGGCAGGAGAGGCAGCTAACGACGCAGCCCGTTTCTTATTTCTGTTTTCTCACAAGCGATGAAAGTGGAAAAGAGGGTGAGCAGGTGGCCCACACATGTGCCTCCAGTGCTGCGGCCCCTCCGGGGACCATCAGCCAGGCCCCGGGGAGGGAGCCAGCCACAGTGTGTCCGGCTCTTCTCTGAAGGGAAGAGAGCCTTGAATAGACTGAAGCGAAGACGGTTCTGCAAGGACAAGGCAGACCGAAGGCATTGGTTTTTTTTTTCAGATAAGGAGAATTAGACTCCCAAGTAGACACCAGAGTCACTGTTTGGTTGGTGGGTGATAGTGGGGTCACAGTGGCTGCCTGTGCTCCCCCAGGGTGAGCGTGACTGTGCTAACCTGGGTGGGGCAGCATGCACACCCCTCTGGCAGCCCTTTGTTGCTCGCTGATGACAAGTTTGGATGATCCCGCCAAACAGCTTGCTAAGATGTAGTCCCCAGTGTTGGAGGTGGGGCCTGATGGGAGGTGCTACCCTTGTGAGATAAGGTTGTGTAAAAGCCTGTGGCACCTCCCCACACTGACGCTCTCACCCCTGCTCTGGCCATGTGCCGCGCCTGCTCCCACTTCCCCTTCTGCCAGGAGTAAAAGCCCCCGAGACCTCCCAGAAGCCAAGCAGATGCTAGTGCCATGCTTCCTCTGCAGCCTGCAGAACTGTGAGCCAATTAAACCTCTTTTCTCTATAAA

**3、The blue fonds were predicted as the promising binding sites of miR-585-5p based on seed sequence alignments. The yellow-highlighted sequences were the top-rated which we chose for follow-up detection.**

| **NCBI Gene ID** | [4286](http://www.ncbi.nlm.nih.gov/entrez/query.fcgi?db=gene&cmd=Retrieve&dopt=full_report&list_uids=4286) | **GenBank Accession** | [NM_000248](http://www.ncbi.nlm.nih.gov/entrez/query.fcgi?cmd=Search&db=Nucleotide&term=NM_000248) |
| --- | --- | --- | --- |
| **Gene Symbol** | MITF | **3' UTR Length** | 3089 |
| **Gene Description** | melanocyte inducing transcription factor | | |

>hg38_knownGene_ENST00000352241.8 range=chr3:69965249-69968336 5'pad=0 3'pad=0 strand=+ repeatMasking=none

CGAATCCTCCCTGCACTGCATTCGCACAAACTGCTTCCTTTCTTGATTCGTAGATTTAATAACTTACCTGAAGGGGTTTTCTTGATAATTTTCCTTTAATATGAAATTTTTTTTCATGCTTTATCAATAGCCCAGGATATATTTTATTTTTAGAATTTTGTGAAACAGACTTGTATATTCTATTTTACAACTACAAATGCCTCCAAAGTATTGTACAAATAAGTGTGCAGTATCTGTGAACTGAATTCACCACAGACTTTAGCTTTCTGAGCAAGAGGATTTTGCGTCAGAGAAATGTCTGTCCATTTTTATTCAGGGGAAACTTGATTTGAGATTTTTATGCCTGTGACTTCCTTGGAAATCAAATGTAAAGTTTAATTGAAAGAATGTAAAGCAACCAAAAAGAAAAAAAAAAAGAAAGAAAGAGGAAAAGAAATCCATACTAACCCTTTTCCATTTTATAAATGTATTGATTCATTGGTACTGCCTTAAAGATACAGTACCCCTCTAGCTTTGTTTAGTCTTTATACTGCAAACTATTTAAAGAAATATGTATTCTGTAAAAGAAAAAAAAAATGCGGCCTTTTCATGAGGATCGTCTGGTTAGAAAACATAACTGATACCAACCGAAACTGAAGGGAGTTAGACCAAGGCTCTGAAATATAAAGTCTAATCTTGCTCTCTTTTATTCTGTGCTGTTACAGTTTTCTTCATCAATGAGTGTGATCCAGTTTTTCATAAGATATTTTATTTTGAAATGGAAATTAATGTCCTCTCAAAGTAAAATATTGAGGAGCACTGAAAGTATGTTTTACTTTTTTTTTATTTTATTTTTGCTTTTGATAAGAAAACCGAACTGGGCATATTTCTAATTGGCTTTACTATTTTTATTTTTAAATTATGTTTTACTGTTCATTTGATTTGTACAGATTCTTTATTATCATTGTTCTTTTCAATATATTTGTATTAATTTGTAAGAATATGCATCTTAAAATGGCAAGTTTTCCATATTTTTACAACTCACTGGTGGTTTTCCGCATTCTTTGTACACCCATGAAAGAAAACTTTTATGCAAGGTCTTGCATTTAAAAGACAGCTTTGCGAATATTTTGTAAATTACAGTCTCACTCAGAACTGTTTTTGGACACATTTAAGGTGTAGTATTAATAGGTTAAAACCAGGCTTTCTAGAAAGAATAAACTTACATATTTATTTTTAGGACATGAAAATAGCAATATTCTTGGAGATTGATAACCATAGCATTAATACGCCCATTATGGTCATTTAAATTGGGGTTTATTTCAGCAAACTTGTTGAATTTATTTTTAAGAAAGAAATACTGTATTGGGAAGTTACTGTTACTTGATAACAATGTTTTAACAAGAAGCAATGTTATAAAGTTAGTTTCAGTGCATTATCTACTTGTGTAGTCCTATGCAATAACAGTAGTGTTACATGTATCAAGCCTAGATGTTTTATACAGATGCCATATAGTGTTATGAGCCAGGCTGTTGAATGGAATTTCTCAGTAGCAGCCTACAACTGAATAGCAAGTGGCATAAAGCATATCCATTCAGAATGAAGTGCCTTAAATATAGCAGTAGTCTTTTTTGGACTAGCACTGACTGAACTGTAATGTAGGGGAAAGTTTCATGATGGTATCTATAGTCAAGACGAACATGTAGCATGGTGCCTATGTAGACAATATAAGAGCTTCCAATTTTCCTTCAGATATTTTTAATATTAAATATATTTTAGTGACAGAGTGCCAACTTCTTTCATCAGGAAACCTTATTCAGGAGGGTTTTTAAAAAGTGTTTAAATGTCAAATGTGAATTGGTGATGGGTGATGGAGGGTTCAGAGAGGAGTGATCGTCAGATGTGTGAATGGACGGTTTAGGTGAAAATAATCAACTGCATAGTTCCCATGCACGCTGGGCAATGAGAATCCTTGGAAACATTGGTGATGCTATCAGTTTTATAGCTTTATTTCTTAAGGGGGTAGGGAAAATTAGTTCCCATTCTTTCAACCCCCTTAACTGTATAGCTCTTTTCCTAGAATAGTGACGCAAATCTGCATGAACAGCTAATTGTACCATAGTGTTCATTGATACAATCATAGCATTGTCTATTTTTCTCTTCATATTTATATGGGGGGGAGGGCGCTGGATGCAAAAGTTGAAGATCGTGATGCTATGATGTTAGTTTTCCTTAGCTGATTTTGAGGGTTTTTAAAAATAAAGCAAGGTTGACTAACCTACGGCCACGGGAACAGGACCATGGTTAAGCAACCATATAGAAAGCTTTGTTGAAAGAAAGTATGGCATCTTGTACCACTGCCCTGACTGTCACAACTCCTAACCTTGCCATTGCCTGCCTCCCCCTCCCCTTCTCCTTAAGAGACAATTTCTGCAGGTGGCAGGTGAGCAAGCCCAGGAGAATGCTGCAATCTTGGGGGTGGTTTTATTTATTTCTTTTTTGCCAAATAGAGTGTGGATTCATTTCAGGGGCTAGCTAAGCCAAGAGGCAGTGGTTTGGGCTTGTTGTTTGTAACAAGAAAATGATCCACACCACTCCCCCGATTCCCGGGTGCAGAATTGTAACTCGGGGTTGGGCCTCTATATGGAGTGACCAAAATGCCAAAATTGTCCATCTGCCTCTGAGTAGGGCAATGGAAATACCAAACCTTCTGACTTTGCCAAAAAGCATACAAGCAACCTGGTCATACATAGGATGACAAAATTCTTTCTGGTTGTTTTTAAACAATAAAGCAATAAGAACAAATACAATACATAGGAAGTTAAAAGCACAAAGGAATGAACTTATTAATATTTTTGAAAAATGCACTGGGAAAAAGTTGATGTCAATAACAGTATAAAACAGCCCTATTTCTTGATAAAAAATGACAAATGACTGTCTCTTGCGGATGCTTGGTACTGTAATGTTAATAATAGTCACCTGCTGTTGGATGCAGCAATAATTTCTGTATGGTCCATAGCACTGTATATTATGGATCGATATTAATGTATCCAATGAAATAATCGACTTGTTCTTGATAGCCTCATTAAAGCATTTGGTTTTTCACATA
